# Is there a difference in sample adequacy when vaginal HPV DNA samples are self-collected at home, a health post, or a primary care health center in rural Ethiopia? Implications for community cervical cancer screening.

**DOI:** 10.1186/s12905-025-04226-9

**Published:** 2026-01-07

**Authors:** Alexandra Hernandez, Sahai Burrowes, Baye Gelaw, Tamrat Abede, Hanamariam Seyoum Alemu, Ayenew Molla Lakew, Yohannes Ayanaw Habitu, Brhanu Teka, Ida Ramezani, Madison Sisk, Buu Dao, Mulat Adefris, Dawit Kassahun, Tiruzer Bekele, Setegn Eshetie, Asmamaw Atnafu, Eiman Mahmoud

**Affiliations:** 1 Public Health Program, College of Education and Health Sciences, Touro University, Vallejo, CA USA; 2 Department of Medicine, University of California, San Francisco, CA USA; 3 Department of Epidemiology and Biostatistics, Institute of Public Health, College of Medicine and Health Sciences, University of Gondar, Gondar, Ethiopia; 4 College of Health Sciences, School of Medicine, Addis Ababa University, Addis Ababa, Ethiopia; 5 Center for Gender Studies, College of Development Studies, Addis Ababa University, Addis Ababa, Ethiopia; 6 Department of Reproductive Health, Institute of Public Health, College of Medicine and Health Sciences, University of Gondar, Gondar, Ethiopia; 7 College of Osteopathic Medicine, Touro University California, Vallejo, CA USA; 8 Department of Gynecology and Obstetrics, College of Medical and Health Sciences, University of Gondar, Gondar, Ethiopia; 9 Department of Pathology, College of Medicine and Health Sciences, University of Gondar, Gondar, Ethiopia; 10 Department of Health Systems and Policy, Institute of Public Health, College of Medicine and Health Sciences, University of Gondar, Gondar, Ethiopia; 11 Department of Medicine, Division of Infectious Diseases, University of California, San Francisco (UCSF), Box 0654, 513 Parnassus Ave, Room S420, San Francisco, CA 94143 USA

**Keywords:** Cervical cancer, Screening, Human papillomavirus, HPV, Self-sampling, Ethiopia

## Abstract

**Background:**

Self-collection of HPV DNA samples is recommended as a cervical cancer screening method in areas with high barriers to clinical examination, such as Ethiopia. Self-collected sample adequacy in clinical settings is high compared to clinician collection, but less is known about self-collected sample adequacy in community settings. We evaluated sample adequacy differences when samples were taken at women’s homes, a local health post, or a primary care health center in rural Ethiopia.

**Methods:**

Women either self-collected vaginal samples for HPV DNA at home (Arm 1, *n* = 100), at a health post (Arm 2, *n* = 100), or at a health center (Arm 3, *n* = 200). Women received identical sample collection kits and illustrated instruction pamphlets; all samples were treated the same way once collected. HPV DNA testing was performed using *Ampfire Multiplex High-Risk HPV (*Atila, Mountain View, California).

**Results:**

Thirty-two (8%) of the 399 samples were inadequate (negative β-globin gene). Sample inadequacy frequency was highest in Arm 1-Home (15%) compared to Arm 2-Health Post (7%) and Arm 3-Health Center (5%) (*p* < 0.05)). Arm 1 had significantly higher odds of inadequacy than Arm 3 (aOR: 2.8, 95% CI 1.2–6.8, *p* = 0.02) when adjusted for age, education, and marital status. There was no difference in adequacy between Arms 2 and 3. HPV prevalence was lowest in Arm 1 (6%) and significantly higher in Arms 2 and 3 (20.4% and 15.9%, respectively, *p* = 0.02). More women in Arm 2 reported positive views of self-sampling, and fewer reported embarrassment when using the self-test compared to Arm 1.

**Conclusions:**

Collecting self-samples at home yielded more inadequate samples than collecting at a health center; however, self-samples collected at the health post were not significantly different from those collected at the health center. In rural areas, self-sampling at locations proximal to women’s residences but providing privacy may increase screening campaigns’ success. While more work is needed to confirm this finding, home sampling may miss women who should be referred for diagnostic testing.

**Supplementary Information:**

The online version contains supplementary material available at 10.1186/s12905-025-04226-9.

## Background

Cervical cancer kills approximately 350,000 women globally, although it is preventable [1]. Low- and middle-income countries (LMIC) account for 90% of the burden of cervical cancer incidence, morbidity, and mortality [2, 3]. In Ethiopia, cervical cancer is the leading cause of cancer-related morbidity and mortality among women, with 7,000 women diagnosed and 5,000 dying annually from the disese [4, 5].

Ethiopia’s national-level initiatives to address cervical cancer include HPV vaccination for girls ages 9 to 13 and a national screening program that uses a “screen and treat” approach with visual inspection with acetic acid (VIA) as the recommended screening method [6–8]. Due to service supply barriers and low demand driven by poor awareness and the fear, embarrassment, and inconvenience involved in screening, few women receive screening before they have symptoms of advanced disease [9–13].

To close this screening gap, researchers and policymakers have encouraged the incorporation of HPV DNA testing for high-risk serotypes into screening protocols using self-collected vaginal or cervical samples [14]. Self-sampling allows women privacy and mitigates the embarrassment and discomfort common in other screening procedures [15, 16]. By triaging the women who need cervical exams, incorporating self-screening into screening protocols could reduce the strain on primary care facilities and mitigate a screening bottleneck.

Numerous studies in SSA, including those in Ethiopia, have demonstrated high acceptability of self-sampling and improvements in follow-up when women have collected their own samples for HPV DNA testing [15, 17]. Self-sampling in LMICs has been shown to have very high sensitivity and specificity, as well as agreement and concordance with physician-collected HPV DNA samples [18–21]. However, until recently, most studies of self-sampling test performance in Ethiopia have evaluated self-sampling in clinical settings [16, 18, 20, 22, 23]. Less is known about the performance of HPV self-sampling conducted at the home or community level. Home- and community-based sampling faces several challenges that require further study. For example, samples collected at home or in the community could be more susceptible to inadequacy from contamination, improper collection procedures, mishandling, and heat exposure than samples taken at health facilities. Previous studies of HPV self-sampling in Ethiopia have found high rates of sample inadequacy, approximately 20% [15, 24]. In addition, there is preliminary evidence from studies of home-based HPV screening in Ethiopia that women with inadequate samples often never receive screening results or outreach for follow-up care and that this gap in care due to inconclusive results leaves them unwilling to continue screening or to recommend screening to others [25].

An important motivation for this study is Ethiopia’s unique health system structure, which makes community- and home-based screening particularly attractive (see Table 1). Screen-and-treat services are currently provided at primary care health centers. However, Ethiopia also has robust village-level primary healthcare services led by health extension workers (HEWs) who provide health education and preventative care at home and village/*kebele*-level health posts. This community-based primary care model has had notable success in expanding access to primary care services in Ethiopia [26, 27], which leads us to ask how HEWs might facilitate HPV-based screening in the home and community.

**Table 1:** Ethiopia’s health system levels and cervical cancer prevention and treatment roles

	Health Posts*	Health Centers	District Hospitals	Referral Hospital	Comprehensive Specialty Hospital
Health System Level	Primary	Primary	Primary	Secondary	Tertiary
Administrative level	Kebele**	*Woreda*/District	Zone	Region	National
Number of People Served	3,000–5,000	Rural: 15,000–25,000 Urban: 40,000	60,000- 100,000	1–1.5.5 million	3.5–5.0.5.0 million
Staffing	Health extension workers (HEWs)***	Midwives, health officers, nurses, laboratory technicians	Advanced practice clinicians (e.g., ob-gyns)	Advanced practice clinicians (e.g., ob-gyns), pathologists, and primary care staff	Oncologists, radiotherapists, pathologists
Current cervical cancer service role	Door-to-door health education, counseling, referrals	VIA, cryotherapy, referrals	VIA, cryotherapy, removal of large lesions, referrals	Diagnosis, staging, removal of large lesions, surgery	Chemotherapy, brachytherapy, radiotherapy, surgery

* A health post is a small 1–2 room building in which health extension workers (HEW) provide services and keep records when they are not providing door-to-door services

** Kebeles are the smallest administrative government unit in Ethiopia. It is the equivalent of a large village, neighborhood, or precinct

*** Health Extension Workers (HEW) are community members who have received at least a grade-ten level of education. They then receive an additional year of training in health promotion, disease prevention, selective curative services, and record keeping. In the field, they provide education, immunizations, family planning and nutrition support, and basic curative services at the community level

While numerous studies have established that self-collection of HPV DNA samples has utility in low-income settings, there remain questions about the technique’s suitability for large-scale utilization in remote rural areas. Few studies in SSA have compared the adequacy of self-collected samples between home and healthcare facilities, and even fewer have compared the adequacy of samples taken in different healthcare settings. Our study contributes to addressing this gap in the literature.

This study compares the adequacy of vaginal self-sample for HPV DNA collected by women receiving the same health education and using identical visual instructions at three locations: the home (Arm 1), their local *kebele* health post (Arm 2), and a local primary care health center (Arm 3). We also determined the type-specific prevalence of cervicovaginal HPV infection overall, and by study arm. Finally, we describe the experiences of the sampling procedure and the comfort of collecting the sample by study arm.

## Methods

### Study setting

**Fig. 1:**
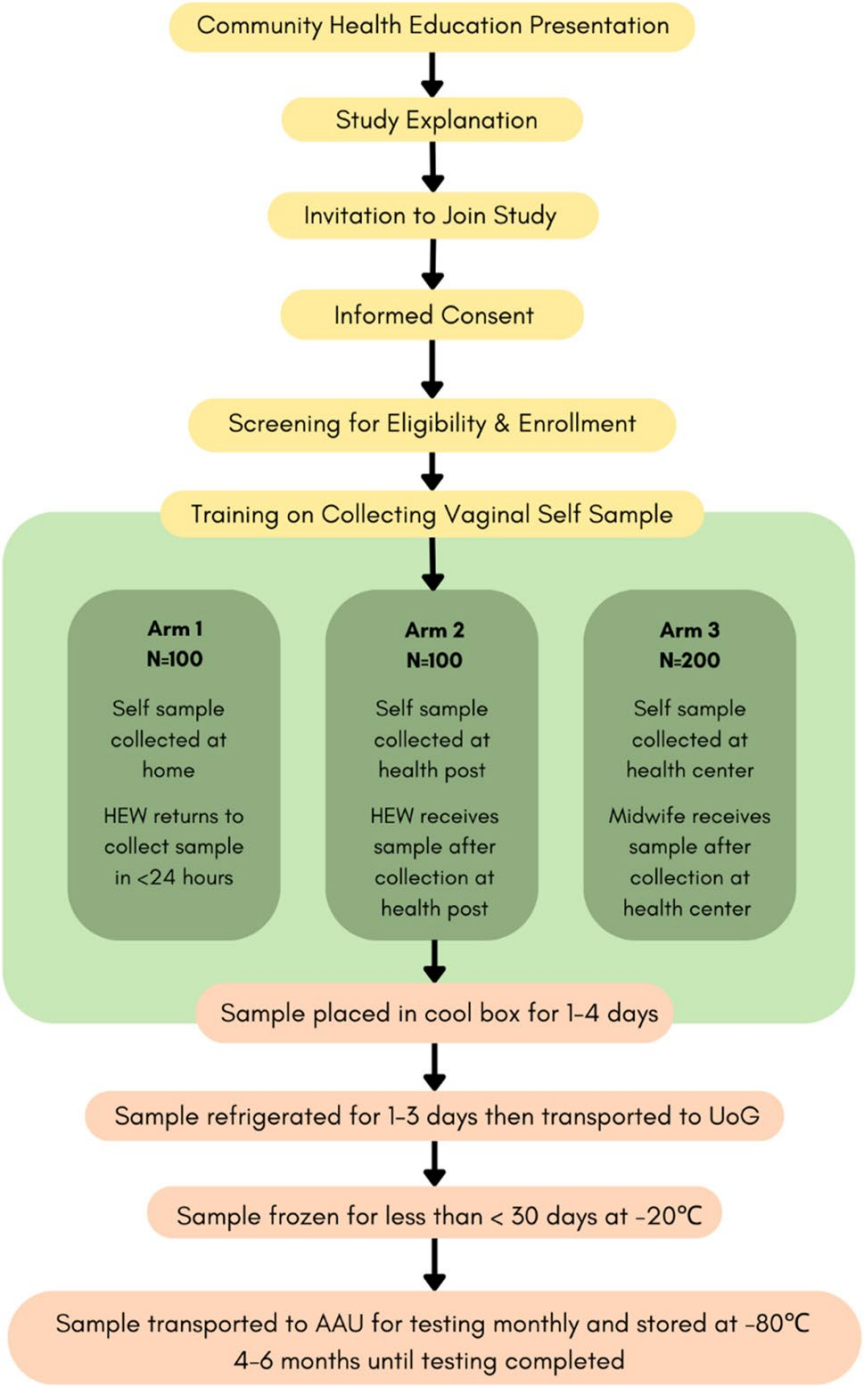
Diagram of the flow of the study. Recruitment and enrollment took place for each arm in the designated Kebele

The project assembled a cross-national team of researchers from Touro University California (TUC), the University of Gondar (UoG), and Addis Ababa University (AAU). Our cross-sectional study was based out of the UoG in the Amhara region of Ethiopia, and women were recruited from three rural *kebeles* in the North Gondar Zone. The study had three arms: Arm 1 (*n* = 100), or the home-based self-sampling arm, took place in the first rural *kebele;* Arm 2 (*n* = 100), or the health post arm, took place in the second; and Arm 3 (*n* = 200 enrolled, 1 participant excluded from analysis for missing HPV test), or the health center arm, in a third (Fig. 1). Data were collected from the 1 st of October 2021 to the 30th of February 2022. Participants for each arm of the study were recruited from a single *kebele* to ensure there was little spillover among the three arms of the study. The three *kebeles* were chosen to have similar demographics and access to health care and were in the catchment area of the health center where the study was conducted. The health center location was chosen based on its proximity to UoG, which has functional VIA screening services and a laboratory. The other two *kebeles* were randomly assigned to the home or health post. At each *kebele*, procedures for recruitment, community education, consent, enrollment, instruction on vaginal self-sampling, and questionnaire administration were identical. Only the procedures for sample collection differed for each arm (Fig. 1).

### Community health education presentation

We developed a 15-minute, Amharic language, illustrated cervical cancer education flip chart for community members based on health education materials developed by the WHO Regional Office for South-East Asia, Grounds for Health, and PATH and Ethiopian Ministry of Health’s guidelines [28–30]. The presentation included drawings that describe basic external and internal female anatomy, basic facts about cervical cancer, the importance of screening for pre-cancer and treatment of precancerous lesions, and information about HPV infection and testing for HPV infection (Supplemental Materials).

### Vaginal self-sample participant training and instruction pamphlet

Investigators jointly developed a flip-chart training for HEWs to deliver to participants on how to collect the vaginal self-sample. We also created an illustrated instruction pamphlet (Supplemental Materials) with drawings of the sample self-collection process based on the test kit package insert (Atila, Mountain View, California).

### Demographic and behavioral survey

Investigators developed a demographic and behavioral survey adapted from the My Body My Test Questionnaire [31]. It queried women about demographic descriptors, reproductive and medical history, knowledge about HPV and cervical cancer, and experiences with collecting the vaginal self-sample. We also included question on methods that could be used for future cervical cancer screening interventions such as comfort shipping samples, or comfort receiving test results by phone. The survey was administered by an interviewer and collected on paper forms after sample collection, and a data clerk at UoG entered the data.

### Piloting methods and focus group discussion

The project held two focus group discussions (FGDs) to pretest our community health education materials (i.e., the flip charts and script), the self-collection instructions presentation and pamphlet, the study questionnaire, and our recruitment materials (Supplemental Materials). Two project HEWs held a mock education session with focus group discussants using the draft materials. Discussants were then requested to collect a vaginal self-sample to test for HPV using the study test kit and then to complete the interviewer-administered questionnaire. The facilitator collected feedback from each respondent on the materials regarding their clarity, points of confusion, and suggestions for improving wording. Project investigators reviewed the translated transcripts for themes and key recommendations and used this information to revise the health education flip charts, questionnaire, and self-sampling instructions. Women who participated in the FGD were not eligible for the main study.

### Recruitment

Recruitment commenced with HEWs advertising cervical cancer health education events to the three *kebeles* using their usual communication channels, such as posting flyers, asking community volunteers to share information, and directly inviting women. On the day of each event, HEWs delivered the cervical cancer health education presentation, responded to questions, and invited women to stay and learn about our study. Women who expressed interest were scheduled for a screening and consent conversation.

### Eligibility and screening

Women were eligible if they were between the ages of 30 and 50 and were residents in a study *kebele*. Women were ineligible if they had previously been screened for cervical cancer through HPV testing, were pregnant, or had had a hysterectomy.

HEWs described the study in detail, reviewed risks and benefits, read the informed consent form, and answered any questions posed by potential participants. Participants in Arm 1 were educated on collecting the vaginal self-sample and given all materials needed for sample collection that day. A time was scheduled for the HEW to return to collect the sample in less than 24 h post-sample collection. Participants in Arms 2 and 3 were scheduled for a visit to collect their samples at the health post or health center and received education on collecting the vaginal self-sample at the time of their visit.

### Vaginal self-sample collection

The sample test collection kit, provided by the test kit manufacturer (*Atila*,* Mountain View*,* California*), contained a zip lock bag, brush, a sample collection tube, and the instruction pamphlet prepared for this study. Women were instructed on how to collect the sample with the flip charts described above. All women were given the visual instruction pamphlet to use during their private sample collection. In Arm 1, they were talked through the procedure, given the kit and instructions, and asked to complete sample collection at home. In Arm 2, women were talked through the procedure by the HEW and asked to take their sample in a screened section of the health post while the HEW waited on the other side of the screen. In Arm 3, women were talked through the procedure by the midlevel provider, and the sample was taken in the exam room while the midlevel provider waited outside the door.

### Sample storage and transportation

In Arms 1 samples were kept at room temperature and picked up by the HEW and delivered to the health post with in 24 h. At the local health post, sample tubes from both Arms 1 and 2 were stored at room temperature until it was time to transport samples to the health center on Friday evenings after a Monday-Friday work week. In Arm 3, samples were also placed in a cold storage box until the end of the week so that samples collected in the three arms had similar times until refrigeration. At the health center, all the samples collected from all study arms were placed in the refrigerator (4 °C) until transportation to UoG within 1 week; at UoG, they were stored at −20 °C for no more than 30 days. UoG laboratory staff packed samples in dry ice for shipment to the Department of Microbiology, UAA, and transported them by air. While we had originally planned to send samples to UAA once a week via a shipping company, disruptions because of COVID and political unrest required us to decrease to once a month for the four-month study period.

### Laboratory testing

HPV DNA testing was performed by Addis Ababa University Microbiology Laboratory using the *Ampfire Multiplex High-Risk HPV by Fluorescent Detection Screening Test (Atila*,* Mountain View*,* California)*, which tests for HPV 16 and HPV 18 individually and a combination of 15 other high-risk HPV types. Samples that tested positive for one of the other 15 high-risk types were genotyped with *Ampfire Genotype 15 High Risk HPV by Fluorescent Detection* (Atila, Mountain View, California), including the following HPV types: 16, 18, 31, 33, 35, 39, 45, 51, 52, 53, 56, 58, 59, 66, 68. Test kits are valid at room temperature for 30 days. Samples were randomized prior to laboratory processing to minimize potential bias related to the collection site. Laboratory technicians were blinded to participant study arm when testing.

### Statistical analysis

We summarized the data from our questionnaire with frequencies and proportions for categorical variables and medians and interquartile range (IQR) for continuous variables. If greater than 20% of participants in a single arm were missing data on a specific questionnaire item, we omitted the item from our analysis. We compared differences among the characteristics of our three study arms using the chi-squared test for independence or the Wilcoxon rank-sum test, as appropriate. Further comparisons were made between Arm 1 and Arm 2, Arm 1 and Arm 3, and Arm 2 and Arm 3. *P*-values less than 0.05 were considered significant differences in a characteristic by the study arm.

A participant’s sample was considered adequate if it was positive for the β-globin housekeeping gene or negative for β-globin but positive for HPV DNA on the HPV screening test. Inadequate samples were negative for HPV DNA on the HPV screening test and negative for β-globin. Proportions of inadequate samples were calculated overall and for each study arm. Overall, Chi-squared tests were used to determine if the proportion of “inadequate” samples varied significantly by study arm (Arm 1, Arm 2, Arm 3). *P*-values less than 0.05 were considered significant. We calculated unadjusted odds ratios (OR) and 95% confidence intervals (CI)s of having an inadequate sample comparing Arm 1 to Arm 2 and Arm 1 to Arm 3 using generalized estimating equations (GEE) with a logit link. We hypothesized that age, education level, and marital status could be associated with the ability to perform the self-collection and the adequacy of the sample and that these demographic factors may have been imbalanced among our three arms; therefore, our a priori analysis plan included adjusting our estimates by age, education level, and marital status.

We also conducted an exploratory analysis to identify potential factors associated with participants who had inadequate samples. We explored individual associations between having an inadequate sample and demographic factors, medical history, and questions that evaluated participant experience with taking a self-sample. For this purpose, we used chi-squared tests and ORs and 95% CI, as described above. Because of the exploratory nature of this analysis, we considered *p*-values less than 0.1 to show a significant association with sample adequacy. We included all factors associated with inadequate samples at the *p* = 0.1 level in a multivariable GEE full model and eliminated variables through stepwise backward elimination, retaining variables at a *p* < 0.1 level.

We calculated the presence of type-specific HPV infection among women with adequate samples overall and by study arm with frequencies and proportions. A woman was considered to have “Any HPV” if she was positive for at least one oncogenic HPV type among the 15 types included in our test. She was considered negative for “Any HPV” if she was negative for all individual HPV types. We included all factors associated with Any HPV at the *p* = 0.1 level in a multivariable GEE full model and eliminated variables through stepwise backward elimination, retaining variables at a *p* = 0.05 level of significance.

### Communication of results and referral for care

Approximately four weeks after the self-tests were conducted, women were visited by the HEW to communicate their results. If their HPV DNA test results were negative, they were told that their results were negative and that no further testing was necessary at this time. If their test results were positive for one or more HPV types, they were told that the screening result indicated that further testing was necessary, and they were referred to UoG for Pap smear and standard of care referrals or treatment as needed. If women’s tests were inadequate, they were told that their test could not be interpreted, and they were also referred to UoG for a Pap smear.

## Results

### Characteristics of study population

We enrolled 400 women: 100 women in Arm 1 (home collection), 100 in Arm 2 (health post), and 200 in Arm 3 (health center) (Table 2). One woman in Arm 3 did not have an HPV DNA sample and was therefore excluded from further analyses (Arm 3 *N* = 199). Women’s ages ranged from 30 to 50, and the mean age was 37 years (interquartile range (IQR) 32–43). Women in Arm 2 were significantly younger than those in Arm 1 or 2 (median age 40, 35, and 37 for Arms 1,2, and 3, respectively, *p* < 0.01). Married women accounted for 98%, 91%, and 85% of Arms 1, 2, and 3 participants, respectively (*p* < 0.01). Roughly 95% of women had children. Most women (78%) had no formal education, and those in Arm 1 had significantly lower levels of education than those in Arms 2 or 3 (*p* < 0.001). Very few women reported ever smoking cigarettes.

**Table 2:** Participant characteristics by study arm

	All Participants		Arm 1 Sampled at Home		Arm 2 Sampled at Health Post		Arm 3 Sampled at Health Center		Overall
Characteristic	*N*	(%*)	*N*	(%*)	*N*	(%*)	*N*	(%)	*P*-value
Total *N*	399	(100)	100	(25.1)	100	(25.1)	199	(49.9)	
Age Categories*†									
30–35	175	(44.0)	34	(34.0)	55	(55.6)	86	(43.2)	**0.0047**
36–40 years	99	(24.9)	22	(22.0)	20	(20.2)	57	(28.6)	
40–50	124	(31.2)	44	(44.0)	24	(24.2)	56	(28.1)	
Age of First Sexual Encounter*†									
Less than 15	217	(57.0)	64	(64.0)	50	(50.5)	103	(56.6)	**0.0016**
15–19 years	125	(32.8)	36	(36.0)	32	(32.3)	57	(31.3)	
Greater than 20 years	39	(10.2)	0	(0)	17	(17.2)	22	(12.1)	
Highest Level of Education*†									
None	310	(77.7)	97	(97)	73	(73.0)	140	(70.4)	**< 0.0001**
Primary or Higher	89	(22.3)	3	(3.0)	27	(27.0)	59	(29.6)	
Married*†	358	(89.7)	98	(98.0)	91	(91.0)	169	(84.9)	**0.0019**
Number of Lifetime Sexual Partners	.		.		.		.		
One	213	(54.2)	59	(59.0)	47	(48.5)	107	(54.6)	0.3978
Two	130	(33.1)	33	(33.0)	34	(35.1)	63	(32.1)	
Three or more	50	(12.7)	8	(8.0)	16	(16.5)	26	(13.3)	
Have had Children‡	384	(96.5)	98	(98.0)	99	(99.0)	187	(94.4)	0.0839
Currently Using Contraception*†	168	(43.6)	75	(75.0)	35	(35.4)	58	(31.2)	**< 0.0001**
Type of Contraception*†‡									**< 0.0001**
Birth control pills	0	(9.4)	5	(6.7)	5	(14.7)	6	(9.8)	
IUD	1	(0.6)	1	(1.3)	0	(0)	0	(0)	
Injectable	131	(77.1)	69	(92.0)	23	(67.6)	39	(63.9)	
Hormonal Patch	15	(8.8)	0	(0)	2	(5.9)	13	(21.3)	
Norplant	4	(2.4)	0	(0)	4	(11.8)	0	(0)	
Tubal ligation	3	(1.8)	0	(0)	0	(0)	3	(4.9)	
Condoms	0	(0)	0	(0)	0	(0)	0	(0)	
Ever smoke cigarettes *‡	4	(1.1)	0	(0)	4	(4.0)	0	(0)	**0.0036**
Past HPV or Cervical Cancer Diagnosis	10	(2.5)	0	(0)	3	(3.1)	7	(3.5)	0.1764
Worried about HPV Infection†‡	147	(43.1)	4	(8.3)	20	(20.2)	123	(63.4)	**< 0.0001**
Worried about Cervical Cancer†‡	157	(39.8)	17	(17.2)	13	(13.3)	127	(64.5)	**< 0.0001**
Past STI Diagnosis†	17	(4.3)	1	(1.0)	3	(3.1)	13	(6.7)	0.0602

The overall *p*-value was generated from the chi-square test for independence for comparing all arms and individual characteristics. We enrolled 400 women: One woman in Arm 3 did not have an HPV DNA sample and was therefore excluded from further analyses. When numbers do not add up to the column total, it is because data were missing

* *p* < 0.05 for comparison between Arm 1 and Arm 2

† *p* < 0.05 for comparison between Arm 1 and Arm 3

‡ *p* < 0.05 for comparison between Arm 2 and Arm 3

Just over half of the women in our sample had been tested for HIV. None reported being diagnosed with HIV, and only 4% (*n* = 17) reported a past diagnosis of a sexually transmitted infection (STI). Women in Arm 3 had a higher proportion of past STIs than in Arm 1 (6.7% v. 1%, *p* = 0.03). The study arms also varied in the proportion that used contraception (75%, 35%, and 31%, for Arms 1,2, and 3, respectively, *p* < 0.0001).The most common contraception reported was injectable contraceptives; Arms 2 and 3 had more variety in the types of contraception used than Arm 1. No women in our study reported using condoms. There was no significant difference in the number of past sexual partners among women in the three arms.

### Experience with self-collection

Women in our study differed in their experience with self vaginal swab sample collection by study arm (Table 3). When asked if they had a positive view of self-collection, only 28% of women in Arm 1 reported a positive experience, compared to 93% in Arm 2 and 64% in Arm 3 (*p* < 0.0001). When asked what emotions or feelings they experienced when collecting their sample, 81% of women in Arm 1 reported being “embarrassed” or ashamed” compared to 43% and 34% in Arms 2 and 3 (*p* < 0.0001). Women in Arm 1 also reported feeling overwhelmed and surprised more frequently than in Arms 2 and 3. However, more women in Arm 3 reported feeling “anxious or worried” (4%, 16%, 28% in Arms1,2, and 3 (< 0.0001), and more in Arm 3 reported being “afraid or fearful”.

**Table 3:** Experience with vaginal self-sample by study arm

	All Participants		Arm 1 Sampled at Home		Arm 2 Sampled at Health Post		Arm 3 Sampled at Health Center		Overall
Characteristic	*N*	(%*)	*N*	(%*)	*N*	(%*)	*N*	(%)	*P*-value
Total *N*	399	(100)	100		100		199		
Had a positive experience taking the test*†‡	238	(62.0)	27	(27.8)	90	(92.8)	121	(63.7)	**< 0.0001**
What emotions or feelings did you have when you used the test?									
Anxious or worried†‡	62	(15.5)	4	(4.0)	9	(9.0)	49	(24.6)	**< 0.0001**
Embarrassed of shame*‡	161	(40.4)	77	(77.0)	25	(25.0)	59	(29.6)	**< 0.0001**
Afraid or fearful*†	127	(31.8)	12	(12.0)	35	(35.0)	80	(40.2)	**< 0.0001**
Relieved	1	(0.3)	0	(0)	0	(0)	1	(0.5)	0.6050
Empowered or confident*‡	8	(2.0)	0	(0)	0	(0)	8	(4.0)	**0.0167**
Overwhelmed*†	34	(8.5)	30	(30.0)	0	(0)	4	(2.0)	**< 0.0001**
Intimidated	5	(1.3)	1	(1.0)	3	(3.0)	1	(0.5)	0.1813
Surprised*†‡	28	(7.0)	23	(23.0)	5	(5.0)	0	(0)	**< 0.0001**
Awkward*‡	12	(3.0)	0	(0)	8	(8.0)	4	(2.0)	**0.0021**
Afraid of health implications of test*‡	100	(25.2)	14	(14.1)	7	(7.1)	79	(39.7)	**< 0.0001**
Concerned others would find out about test results	8	(2.0)	1	(1.0)	2	(2.0)	5	(2.5)	0.6793
Thinks the HPV self-test is safe*	308	(78.0)	91	(91.9)	79	(81.4)	138	(69.3)	**< 0.0001**
Feels comfortable shipping sample	388	(97.5)	100	(100.0)	96	(97.0)	192	(96.5)	0.1740
Concerned sample will end up in the wrong hands	21	(5.3)	8	(8.0)	4	4 (4.0)	9	(4.5)	0.3700
Comfortable getting results by phone†	308	(78.0)	91	(91.9)	79	(81.4)	138	(69.3)	0.1580

The overall *p*-value was generated from the chi-square test for independence for comparing all arms and individual characteristics. We enrolled 400 women: One woman in Arm 3 did not have an HPV DNA sample and was therefore excluded from further analyses. When numbers do not add up to the column total, it is because data were missing

* *p* < 0.05 for comparison between Arm 1 and Arm 2

† *p* < 0.05 for comparison between Arm 1 and Arm 3

‡ *p* < 0.05 for comparison between Arm 2 and Arm 3

Most women in the study reported believing that the HPV self-collection was safe; they were not concerned that others would find out the test results and were not concerned that the sample would end up in the wrong hands. These results did not differ by arm. Most women also felt comfortable shipping their samples and receiving results by phone (Table 3).

### Adequacy of self-collected samples

**Fig. 2:**
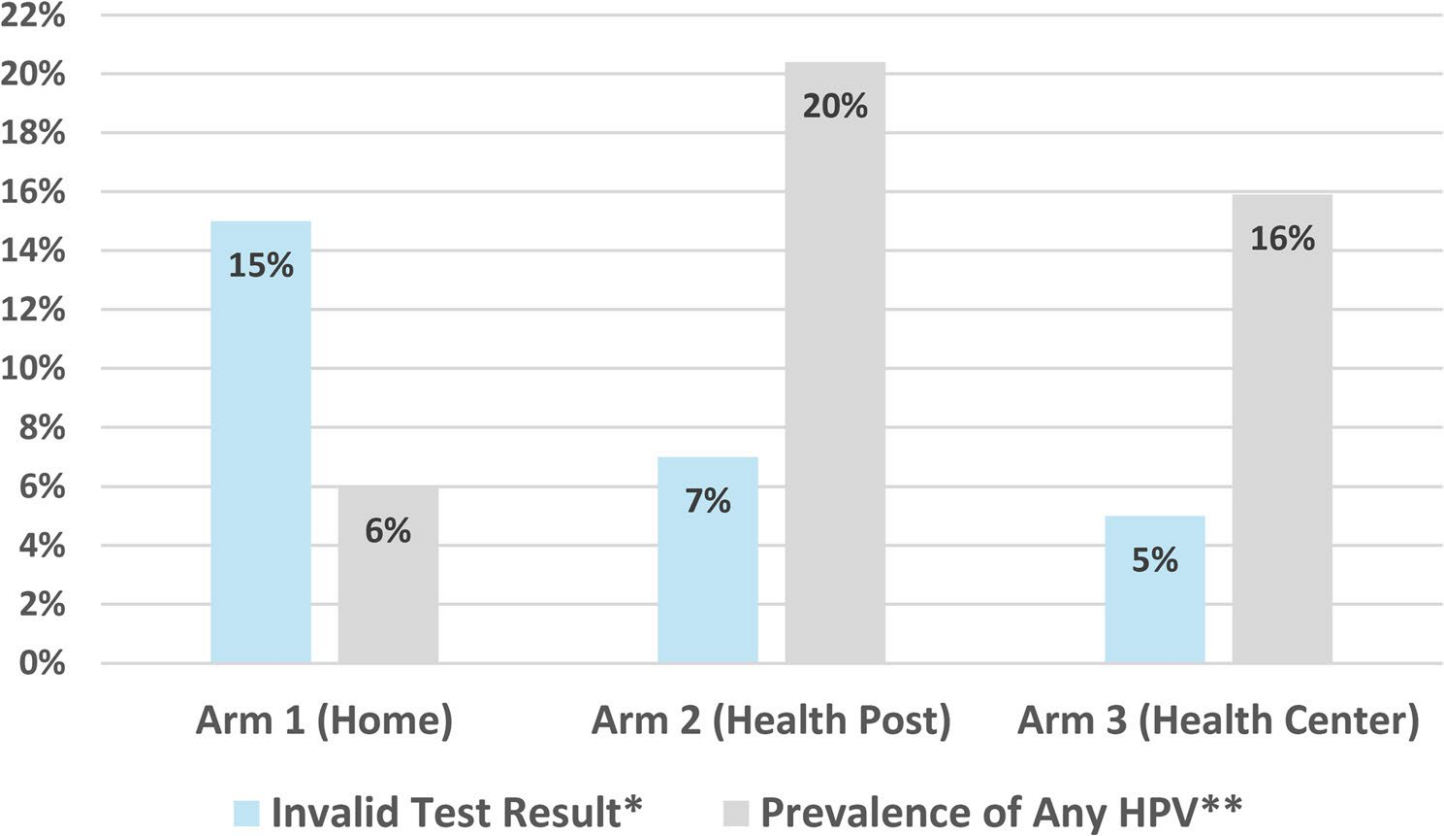
Proportion of invalid test results (blue) by location (study arm) of self-collected vaginal HPV DNA sample and prevalence of any HPV DNA detected in valid samples by study arm. Arm 1 *N*=100 for test adequacy and *N*=85 for HPV prevalence. Arm 2 *N*=100 for test adequacy and *N*=93 for HPV prevalence. Arm 3 *N*=199 for test adequacy and *N*=189 for HPV prevalence. *P-value=0.01 for overall chis-square; *p*=0.07 for comparison of Arm 1 to Arm 2; *p*=0.003 for comparison of Arm 1 to Arm 3. ** *P*= 0.02 for overall chi-square; *p*=0.005 for comparison of Arm 1 to Arm 2; *p*=0.02 for comparison of Arm 1 to Arm 3

Of the 399 samples collected, 32 were found to be inadequate by our laboratory (Fig. 2). Fifteen samples (15%) were inadequate from Arm 1, seven samples (7%) were inadequate from Arm 2, and 10 samples (5%) were inadequate from Arm 3 (Fig. 2). In unadjusted analyses, participants in Arm 1 had 2.3 times the odds of having an inadequate test compared to Arm 2 and 3.3 times compared to Arm 3. In adjusted models, the increase in odds for Arm 1 was only significant when compared to Arm 3 (OR: 2.8, 95% CI 1.16, *p* = 0.02) when adjusted for age, education, and marital status. There was no significant difference in sample adequacy between Arm 2 and Arm 3 (Table 4).

**Table 4:** Comparison of inadequate test results among study arms, unadjusted and adjusted results (*N* = 399)

	Unadjusted			Adjusted		
Characteristic	OR	95% CI	*P*-value	OR	95% CI`	*p*-value
Study Arm						
1 vs. 2	2.34	(0.91–6.02)	0.0769	2.06	(0.78–5.48)	0.1470
1 vs. 3	3.34	(1.44–7.72)	0.0050	2.80	(1.16–6.77)	0.0222
2 vs. 3	1.42	(0.52–3.86)	0.4885	1.36	(0.50–3.70)	0.5500
Age (1 year increase)	1.00	(0.95–1.06)	0.9634	0.99	(0.94–1.05)	0.8237
Primary or Higher Education	0.47	(0.16–1.39)	0.1736	0.66	(0.21–2.08)	0.4696
Married	0.26	(0.04–1.98)	0.1955	0.37	(0.05–2.91)	0.3468

In our exploratory analysis, currently using contraceptives (aOR: 2.0, 95% CI: 1.0–4.2.0.2) and reporting the emotion of “embarrassment” (aOR: 2.1, 95% CI 0.9–4.8) were the only two factors analyzed that showed association with an invalid test at the 0.1 *p*-value level. Age, education, marital status, having children, and number of past sexual partners did not show an association with having an inadequate test.

### Prevalence of HPV

**Fig. 3:**
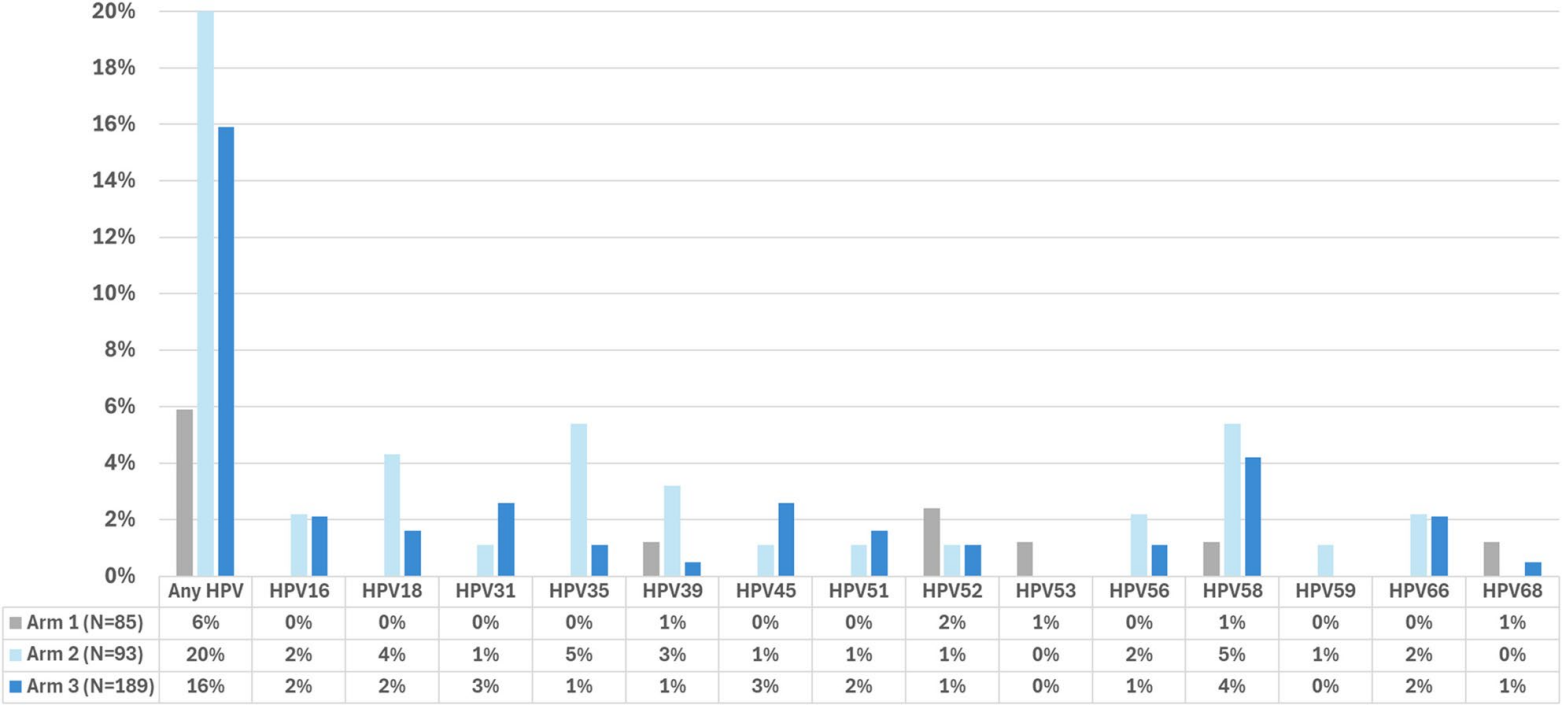
Type-specific prevalence of HPV DNA detected from valid cervicovaginal self-collected samples by study arm. Arm 1 collected at home, Arm 2 collected at the local Health Post, Arm 3 collected at the regional health center

The overall prevalence of HPV infection among women with valid samples (*N* = 367) was 14.7% (*n* = 54). There was a significant difference in the prevalence of HPV among our three arms. Five participants in Arm 1 (6%) were HPV-positive, 19 in Arm 2 (20%), and 30 in Arm 3 (16%) (*p* < 0.01) (Fig. 2). The HPV types with the highest prevalence were HPV35 (1.8%) and HPV58 (3.5%) (Fig. 3). The only HPV type that showed a statistically significant difference among the three arms was HPV35, with 0%, 5%, and 1% in Arms 1, 2, and 3, respectively (*p* = 0.02). In our final multivariable model (Table 5), being married (aOR: 2.2, 95% CI 1.0–5.0), having two past sexual partners compared to one partner (aOR: 1.93, 95% CI 1.0–3.8.0.8), and having three sexual partners compared to one partner (aOR: 3.5, 95% CI 1.5–8.1) increased odds of having HPV. Reporting receiving some formal education compared with having no education decreased the odds of having a positive HPV DNA test (aOR: 0.4, 95% CI 0.2–0.7).

**Table 5:** Associations with HPV infection (*N* = 367)

	Unadjusted			Adjusted		
Characteristic	OR	95% CI	*P*-value	OR	95% CI`	*p*-value
Age	1.01	(0.96–1.05)	0.7680	1.01	(0.96–1.06)	0.6105
Primary or Higher Education	2.48	(1.34–4.57)	0.0037	0.37	(0.19–0.72)	0.0038
Married	2.51	(1.17–5.38)	0.0186	2.21	(0.98–4.97)	0.0547
Number of Lifetime Sexual Partners						
1 or less	1.00			1.00		
2	1.99	(1.03–3.85)	0.0414	1.93	(0.98–3.79)	0.0558
3 or more	3.18	(1.42–7.10)	0.0048	3.51	(1.53–8.09)	0.0032
Age of First Sex	1.04	(0.95–1.14)	0.3509	-		
Have Had Children	3.04	(0.88–10.47)	0.0781	-		
Currently Using Contraception	1.72	(0.91–3.24)	0.0929	-		

## Discussion

Our study found that the occurrence of inadequate vaginal HPV DNA samples was increased when self-collection took place in a woman’s home in rural Ethiopia (15%) compared to self-collection occurring in a village health post (7%) or a local health center (5%). We found no significant increase in inadequate sample occurrence between the health post and the health center arms. Our study ensured that health education, recruitment, self-sampling training, self-sampling materials, sample storage, and sample transport were uniform among the three study arms.

There are several reasons why home-based sample collection may have yielded more frequent inadequate samples than testing at a facility. While we took great care in developing, translating, pilot-testing, and revising our education and training materials, there may have been confusion about collecting the sample accurately. Because women in Arm 1 may have taken their sample several hours after they received the sample collection presentation from HEWs (compared to minutes in Arm 2 and Arm 3), some of the instructions may have been forgotten by the time that Arm 1 women took the sample. At home, no one was on hand to answer questions if they arose, while at the health post or health center, staff were present in case questions arose. Other studies that have evaluated sample collection have reported uncertainty and questions when performing self-sampling [25]. Having trained assistance on hand during sample collection may have resolved questions and resulted in more valid tests for Arms 2 and 3. We cannot rule out that there were individual differences in skill, personality or rapport between HEWs, as there would be if self-sampling with HEW support were to be adopted as the standard cervical cancer screening method in Ethiopia. This may have impacted our results, if, for example, HEWs in Arms 2 and 3 were friendlier than Arm 1. However, Arm 1 and Arm 3 had the least contact with HEWs, Arm 1 self-collected alone and Arm 3 self-collected with a midwife/medical officer on hand. Having a competent and approachable HEW at the Health Post could be a focus of a future intervention.

While there was some demographic imbalance among the three groups, in adjusted models, women sampling at home were still more likely to have invalid test results than those in Arm 3, health center. In exploratory analyses, only contraceptive use and feeling embarrassed showed significant association with invalid test results. Most women reported using injectable contraceptives. It is unclear if the relatively high proportion of women using this contraceptive method in Arm 1 could be related to the high proportion of invalid test results in that arm. Women in Arm 1 also reported higher levels of embarrassment than the other arms, which could lead to poor collection technique. This potential association suggests that educators might improve patient experiences and screening efficacy by developing techniques to lessen embarrassment and normalize self-sampling as part of cervical cancer screening. These associations should be tested in a more extensive study.

The proportion of inadequate samples in our home-based self-sampling arm (Arm 1, 15%) was lower than those found in similar, recent Ethiopian studies. For example, another study evaluating home-based HPV DNA cervical cancer screening in rural Gondar found that 25% of their samples were insufficient (inadequate) for testing (on their initial sample) [24]. A randomized control trial comparing the uptake of cervical cancer screening in Ethiopia found that 19.2% of their samples were inadequate for HPV DNA testing [15]. It is difficult to directly compare these results to ours as the studies had different training, recruiting, and data collection protocols and used a different HPV DNA sample collection kit and testing platforms. However, if other screening programs have higher rates of inadequate samples than our study, our conservative results may point to an even larger number of women who are not receiving appropriate screening when sample collection happens at home. A randomized controlled trial that compares women doing home-based self-collection to health post self-collection and that also does HPV sample collection with both sets of women at a health center could clarify discrepancies in inadequacy estimates.

Arm 1 had both the highest occurrence of invalid tests and, among the adequate samples, the lowest prevalence of HPV infection. Arm 1 had only a 6% prevalence compared to 20% and 16% in Arms 2 and 3. Another study of home-based HPV DNA testing from Gondar showed a prevalence of 13.5% [24], more similar to our other two arms. Other studies in Ethiopia have found that the prevalence of HPV ranges from 13.1% − 23.9% [32–35]. The prevalence differences by arm in our study could be explained by the demographics of women in Arm 1: they were older, fewer were unmarried, and fewer had multiple sex partners than women in other study arms. It may also be that women who were able/willing to attend a visit to a health post or health clinic were more likely to have existing gynecological issues and may be at higher risk of STIs. Similarly, in a Kenyan randomized control trial comparing the uptake of cervical cancer screening when testing was offered via self-collection at health tents in the community or standard of care in government health facilities, there was also a statistically higher prevalence of HPV in women who were offered screening at the government health facility [36].

Another potential explanation is that the prevalence of HPV was the same in Arm 1 as in the other arms but that inadequate samples were mostly HPV-positive. Women who were HPV-positive could have had a more difficult time obtaining an adequate sample, for example, if they also had other STIs and had inflammation or had another reason to experience pain with sampling. Women sampling with assistance may have had an easier time if in pain because they could communicate it to a provider. Another possibility is that an undocumented factor in sample collection, storage, or transport puts home-based samples at increased risk of becoming invalid if HPV-positive. Regardless of the reason for the clustering of low prevalence and high inadequacy in Arm 1, if inadequate samples were masking positive test results, it would be important to move community-level self-sampling away from homes and into health posts or other locations with provider support. This finding and hypotheses should be explored in more detail in future studies that include resampling of inadequate samples.

Our findings suggest that health posts may be an ideal location for HPV-based cervical cancer screening in rural Ethiopia. We found only minor differences in the proportion of adequate test results in samples taken at the health post and those taken at the health center. Because health posts are usually closer to women’s homes than health centers, self-collection at health posts could increase screening convenience while mitigating the workload impact on overstretched mid-level providers at health centers. Allowing women to schedule self-sampling appointments at health posts could also address difficulties women may have in finding a time and private place to take their samples in their homes.

In our study, women who self-sampled at home reported feeling overwhelmed, embarrassed and surprised more often than women in the other two self-sampling settings. Apart from answering questions, having a trained professional provide emotional support and reassurance that they are collecting the sample correctly may improve the technique and result in a better sample. While women in high-income settings show a clear preference for home-based sample collection, in low-income settings, the picture is mixed, with several studies finding that women prefer healthcare facilities as a sampling site and that they place high value on having a trusted healthcare provider to answer questions and reassure [37–39]. Future investigations could include qualitative methods that seek to understand why self-collecting at home was less favored in this population. In contrast, more women in Arm 3 reported feeling afraid health implication of the test and fewer thought that the HPV self-test was safe. Women who attended the health center may have seen different messaging in the clinic, communicated with other medical professionals during their visit, or had other experiences that may have impacted their feelings about HPV testing or cervical cancer. Although it was not within the scope of our study, qualitative studies including office audits and in-depth interviews with women could lead to better understanding of this finding.

Limitations of note in our study are as follows. Our study had a modest sample size, which, while acceptable for assessing differences in test adequacy between study arms, may have left us underpowered to conduct stratified analyses of factors associated with sample adequacy and HPV infection. More importantly, because we only had a single *kebele* and data collection team per study arm, differences in our outcomes by study arm may be driven by specific, unobserved *kebele* or data collector differences rather than general differences in the characteristics of the self-sampling locations. While we thoroughly tested our questionnaire questions for comprehension and clarity, many questions resulted in many missing responses because women did not understand what was being asked, and we were forced to drop these questions from our analysis. We could not evaluate or incorporate several questions into our explanatory models because of unclear or missing data. In addition, as our study was localized to three *kebeles* in Gondar, Ethiopia, there are limitations our ability to generalize findings to other regions in Ethiopia and East Africa that have different demographics, cultural norms, attitudes around self-sampling, and access to health care. Finally, our study data collection took place during the COVID-19 pandemic and a period of political turmoil in our study region, which disrupted transportation and led to delays in sample shipment, which may have affected sample quality.

## Conclusions

Despite our limitations, our study has several strengths, the most notable of which is that it is the first study to our knowledge to examine test adequacy in different self-sampling contexts in Ethiopia. Based on our study’s results, we suggest basing HPV DNA self-sampling initiatives in rural Ethiopia at health posts to provide a convenient location while ensuring that well-trained HEWs are on hand to answer questions and counsel women who have uncomfortable emotional responses. We would encourage larger future studies to examine self-sampling at more varied geographic locations and settings in Ethiopia. We would also recommend further studies on procedures to improve the adequacy of samples in rural settings.

## Supplementary Information

Supplementary Materials

## Data Availability

The corresponding author may be contacted for reasonable data requests. Data sets collected and analyzed are not publicly available due to restrictions of our Institutional Review Boards.

## Abbreviations

aOR: Adjusted odds ratios
CI: Confidence interval
DNA: Deoxyribonucleic acid
FGD: Focus group discussion
GEE: Generalized estimating equations
HEW: Health extension worker
HIV: Human Immunodeficiency virus
HPV: Human papilloma virus
IQR: Interquartile range
LMIC: Low and middle-income country
OBGYN: Obstetrician-gynecologist
OR: Odds ratio
SSA: Sub-Saharan Africa
STI: Sexually transmitted infection
TASH: Tikur Anbessa (Black Lion) Specialized Hospital
UoG: University of Gondar
VIA: Visual inspection of the cervix with acetic acid
WHO: World Health Organization
