# Supplementary material for: Is there a difference in sample adequacy when vaginal HPV DNA samples are self-collected at home, a health post, or a primary care health center in rural Ethiopia? Implications for community cervical cancer screening
Source: BMC Womens Health. 2026 Jan 7;26:155. doi: 10.1186/s12905-025-04226-9 (PMC12977413; doi:10.1186/s12905-025-04226-9)
Supplement: Supplementary file 1 — Supplementary Materials [file 12905_2025_4226_MOESM1_ESM.pdf]

APPENDIX L: FOCUS GROUP GUIDES

Focus Group Type A Questions: Women

1. What is conducting the self-test like?
2. What did you like about the self-test?
3. What did you NOT like about the self-test?
4. How did you feel about giving your samples to HEWs
   a. How concerned were you about privacy?
   b. How worried were you about your samples falling into the wrong hands
5. Where did you take the test?
   a. What did you feel about taking the test here?
   b. Where would you have preferred to take the test?
6. If you had a positive HPV screening test result what would stop you from following up to get further tests?
7. If you had to go to the health care facility to follow up reproductive health problem, what would be the challenges?
8. Which kind of health care providers (nurse, midwife, HEW, doctor) would you feel most comfortable speaking with about a reproductive health problem?
9. Has any of you ever had to go to a big hospital for care? Tell me about your experiences there?
   a. What was good?
   b. What was challenging?
10. How do you prefer to communicate with providers (phone, visit clinic, home visit)
11. How would you encourage other women to get screen for cervical cancer with the HPV self-test?
12. What would you improve about how your self-testing was done?

Closing

We are now approaching the end of our discussion. Is there anything else anyone would like to add about cervical cancer care we have not talked about?

- Summarise
- Thank participants
- Collect participant demographic details

እናቶቻችን እና ሴት ልጆቻችን በማህፀን በር ጫፍ ካንሰር እንዳይጠቁ መከላከል

የማህበረሰብ ትምህርት መርጃ ቻርት

ስም፡_______________________________

ቀበሌ፡_______________________________

ይህ መመሪያ ግራውንድስ ፎር ሄልዝስ ወይንም የጤና መሠረቶች በተባለው የማህበረሰብ ትምህርት መርጃ መፅሀፍ ላይ በመመርኮዝ በጎንደር ዩንቨርስቲ እና ቶሮ ዩኒቨርሲቲ ካሊፎርኒያ በትብብር እየተደረገ በሚገኘው **እናቶቻችን እና ሴት ልጆቻችንን በማህፀን በር ጫፍ ካንሰር እንዳይጠቁ መከላከል** ለተባለው ፕሮጀክት የተዘጋጀ ነው፡፡

ባለቤትነቱ 2019 የጎንደር ዩንቨርስቲ እና የቶሮ ዩኒቨርሲቲ ካሊፎርኒያ ነው፡፡ ሁሉም መብቶች የተጠበቁ ናቸው፡፡ ጎንደር ዩንቨርስቲ እና ቶሮ ዩኒቨርሲቲ ካሊፎርኒያ በዚህ መመሪያ ውስጥ የሚገኙ መረጃዎችን ከመጠቀም ጋር የተገናኙ ምንም አይነት ሀላፊነቶችን አይወስዱም፡፡

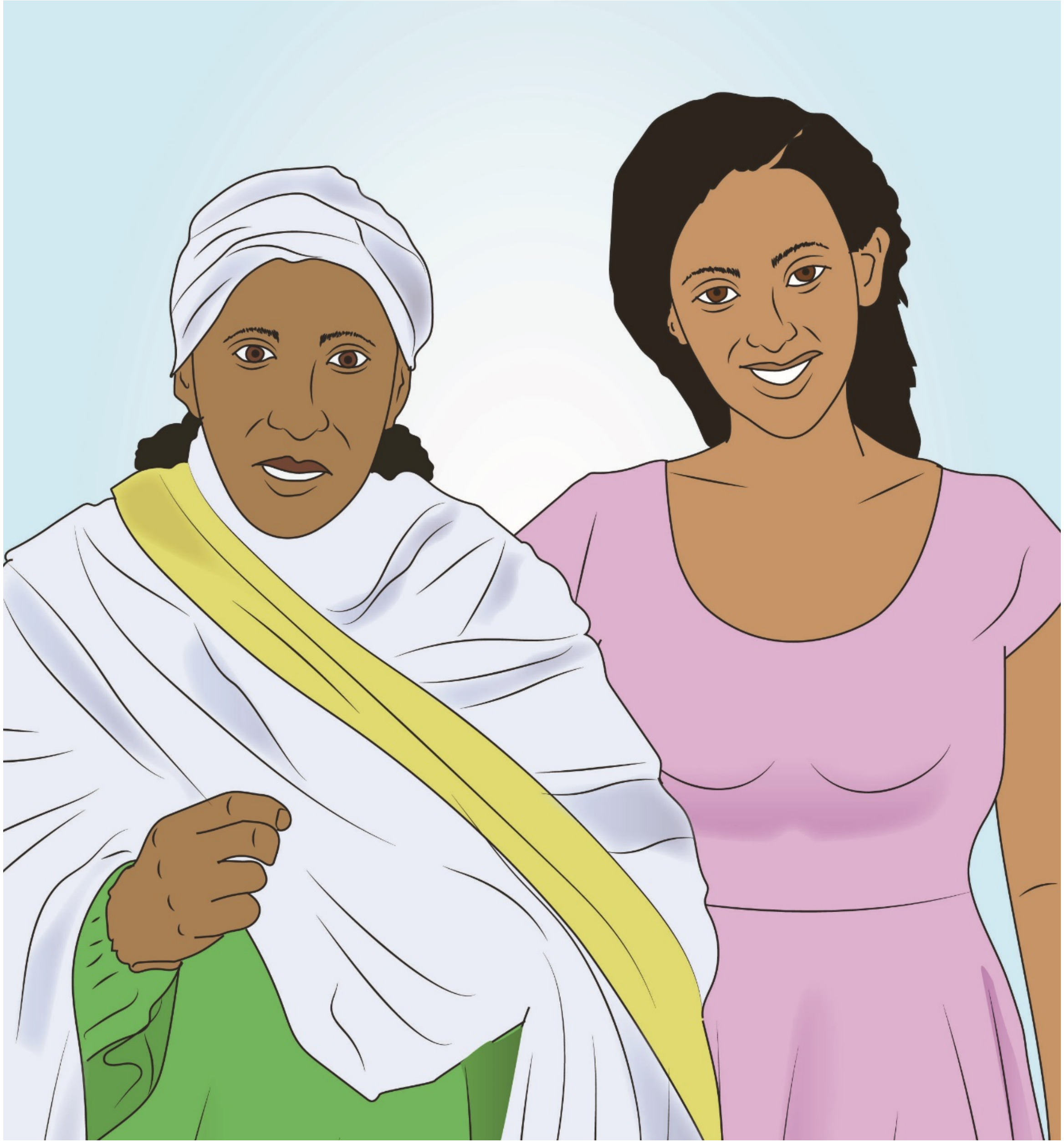

በዚህ ምስል ላይ ምን ይመለከታሉ?

እነዚህ የምትመለከትዋቸው ሴቶች ጤናማ ይመስላሉ?

- በዚህ ምስል ላይ ሁለት ጤናማ እና ደስተኛ የሚመስሉ ሴቶችን እንመለከታለን፡፡
- በህይወት ዘመናችን ጤናችንን ለመጠበቅ ልናደርጋቸው የሚገቡን በርካታ ነገሮች ይኖራሉ፡፡
- የማህፀን በር ጫፍ ካንሰር ማንኛዋንም ሴት ሊይዛት የሚችል በሽታ ነው፡፡ ሁሉም ሴቶች ለዚህ በሽታ ተጋላጭ ናቸው፡፡
- ደግነቱ ይህ በሽታ በሰውነታችን ውስጥ መኖሩ በጊዜ ከታወቀ በሰውነታችን ውስጥ እንዳይስፋፋ መከላከል ይቻላል፡፡

የማህፀን በር ጫፍ ካንሰር የማህፀን በር ጫፍን የሚጠቃ የካንሰር አይነት ነው፡፡

ስለዚህ አሁን ደግሞ የማህፀን በር ጫፍ በአካላችን ውስጥ የት እነደሚገኝ እንነጋገራለን፡-

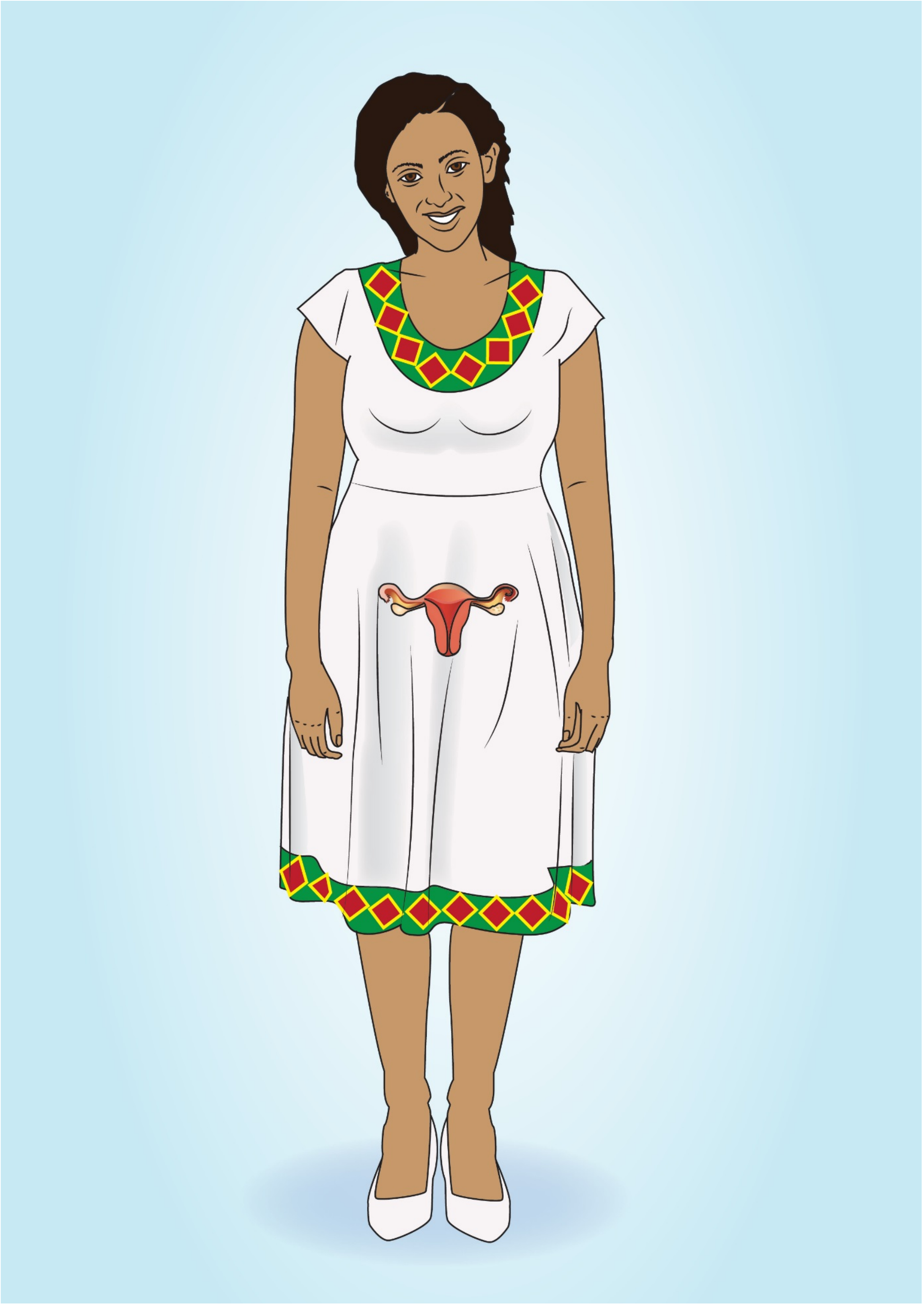

በዚህ ምስል ላይ ምን ይመለከታሉ?

በውስጣችን የሚገኙ ነገር ግን በዐይናችን ልናያቸው የማንችላቸው የሰውነት ክፍሎች እንዳሉን ያውቃሉ?

- የምትመለከቱት ምስል የአንዲት ሴትን ውጫዊ ገፅታ እነዲሁም ከፊት ለፊት በሰውነተዋ ዉስጥ የሚገኙትን የሰዉነት ክፍሎች ማለትም ማህፀንዋን፤ የማህፀንዋን በር ጫፍ እነዲሁም ብልቷን ትመለከታላችሁ፡፡

- ማህፀን የተባለው የሰዉነት ክፍል በእረግዝና ወቅት ልጅ የሚቀመጥበት እና የሚያድግበት የሰውነት ክፍል ነው፡፡

አሁን ደግሞ እነዚህን የውስጥ የሰውነት ክፍሎች ቀረብ አድርገን እንመለከታለን፡-

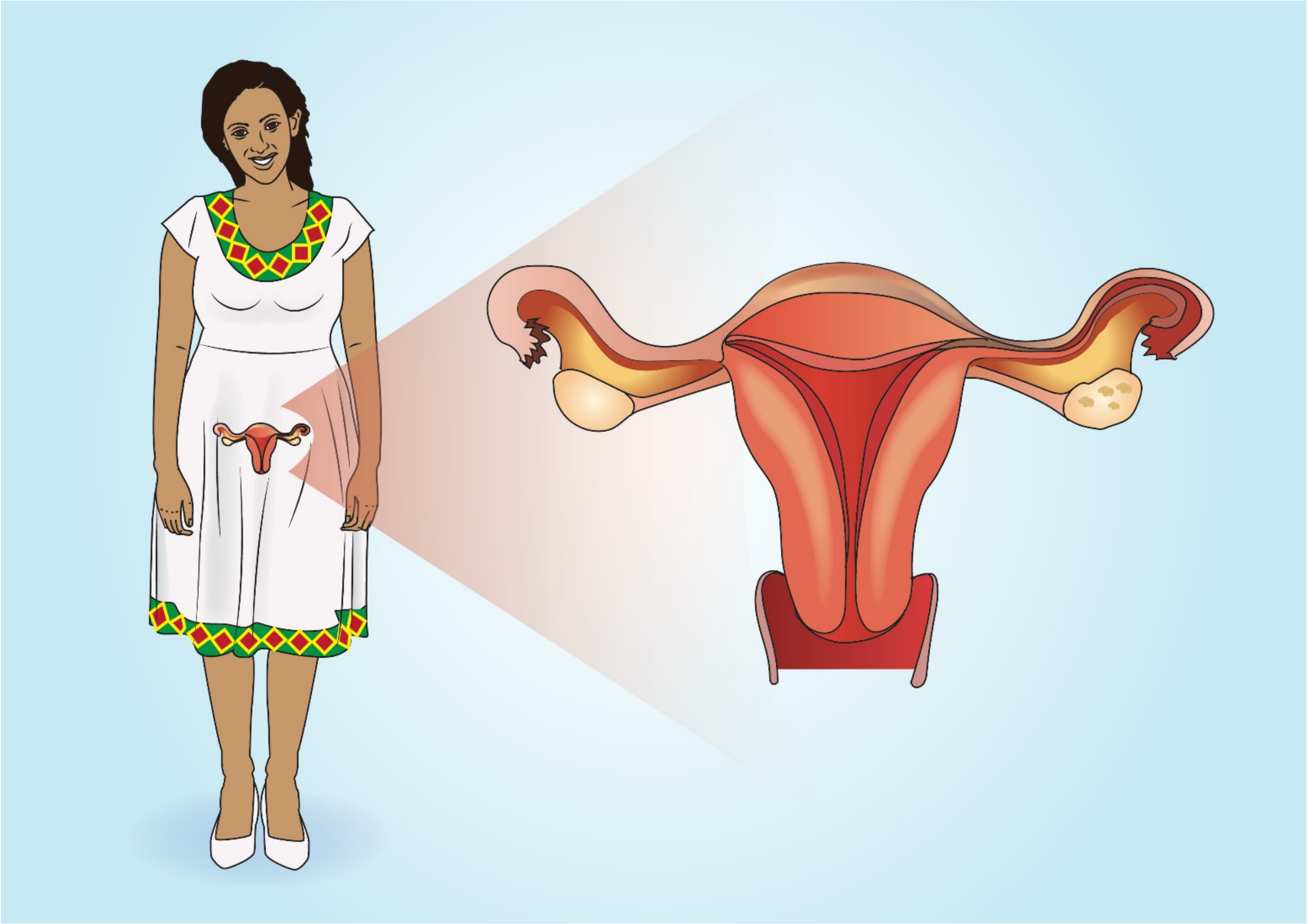

ስዕል ላይ የሚገቡ (to be added to image after approval)

ፋሎፒያን ቲዩብ- የማህፀን ቱቦ

ኦቫሪ- እንቁል እጢ /እንቁላል የሚያመነጭ የሴት አካል

ቫጃይና- የሴት ብልት

ዩትረስ- ማህፀን

ሰርቬክስ- የማህፀን በር ጫፍ

አሁን ደግሞ የማህፀን በር ጫፍ የት እንደሚገኝ እንነጋገራለን

በዚህ ምስል ላይ ምን ይመለከታሉ?

ከመካከላችን ማህፀን የቱ ጋር እንደሚገኝ ሊነግረን የሚችል አለ? የሴት ብልትስ የቱ ጋር ነው የሚገኘው?

- ማህፀን የሚባለዉ መካከል ላይ የሚገኘው ሲን ይህም ልጅ የሚያድግበት የሰውነት ክፍል ነው፡፡ (እያንዳነዱን የሰውነት ክፍል ስማቸውን በመናገር ይጠቁሙባቸው)
- የሴት ብልት የሚገኘው ከማህፀን በታች ነው፡፡
- የማህፀን በር ጫፍ ደግሞ ከማህፀን መጨረሻ ጀምሮ ወደ ብልት የሚወርደው ክፍል ነው፡፡
- የማህፀን በር ጫፍ ወደማህፀን ያለው ክፍተት ነው፡፡

አሁን ደግሞ ይህንን የሰውነት ክፍል የበለጠ ቀረብ አድርገን እንመለከታለን፡-

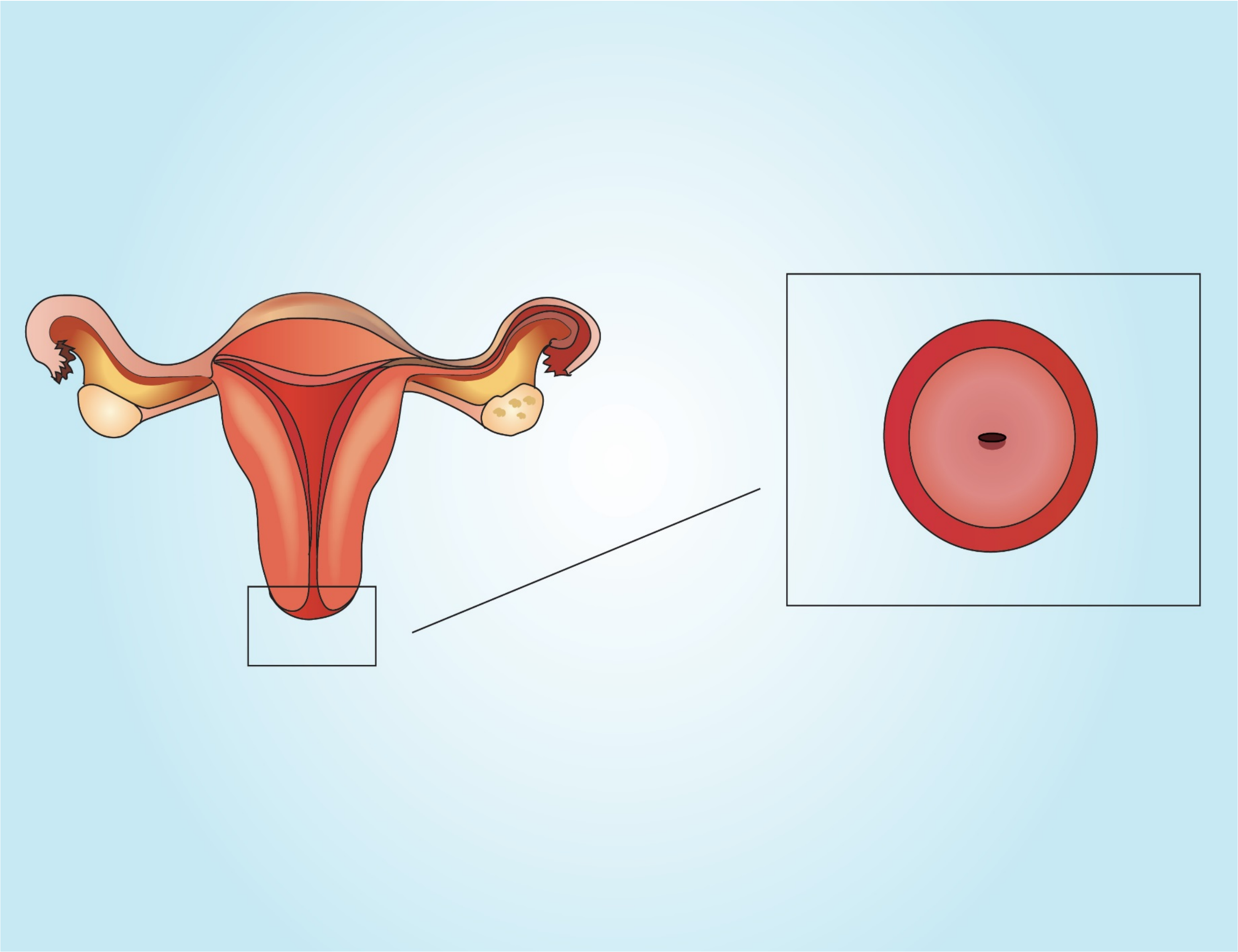

(This should take the entire page!)

በዚህ ምስል ላይ ምን እየተመለከትን እንደነ መናገር ይችላሉን?

- ይህ የምንመለከተው የማህፀን ምስል ነው፡፡ከስር በኩል የምንመለከተው ምስል ደግሞ የማህፀን በር ጫፍ ነው፡፡ *(በቃላት የምንገልፃቸውን የሰውነት ክፍሎች እየጠቆምን እናመልከት)*

- እዚህ ጋር ማስታወስ የሚኖርብን የማህፀን በር ጫፍ ማለት ከብልት እስከ ማህፀን ያለው ክፍተት መኑን ነው፡፡

- የማህፀን በር ጫፍ፤ የማህፀን ካንሰር የሚጀምርበት የሰውነት ክፍል ነው፡፡

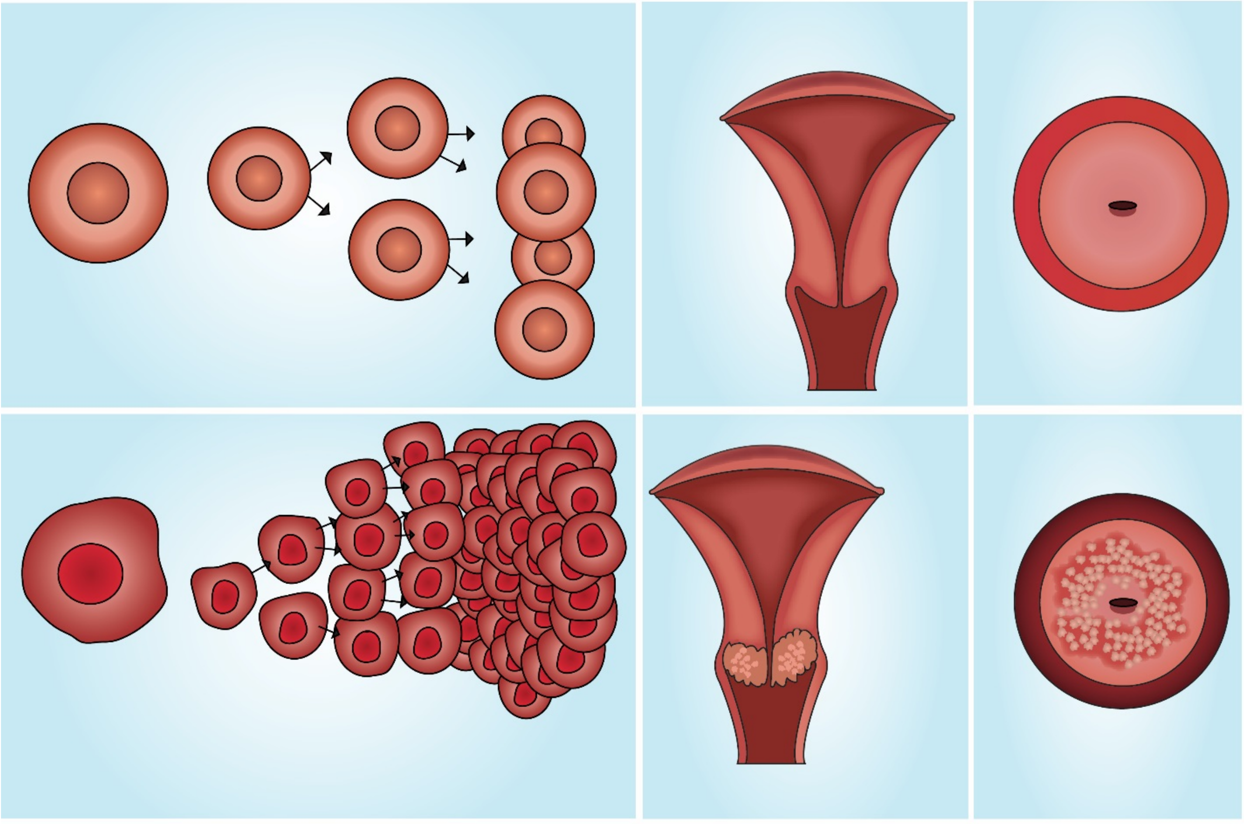

ስዕል ላይ የሚገቡ (to be added to image after approval)

1. ጤናማ የነ የህዋስ መከፋፈል
2. ማህፀን፤ የማህፀን በር ጫፍ፡ ብልት
3. ጤናማ የማህፀን በር ጫፍ
4. የካንሰር ህዋስ ክፍፍል
5. ማህፀን፤ የማህፀን በር ጫፍ፡ ብልት
6. የማህፀን በር ጫፍ ካንሰር

የማህፀን በር ጫፍ ካንሰር ምንድን ነው?

- ካንሰር የተባለው በሽታ የሚከሰተው የሰውነት ህዋሰት ጤናማ ባልነ መልኩ ከቁጥጥር ዉጪ በሚባዙበት ወቅት ነው፡፡
- ካንሰር በማህፀን በር ጫፍ ላይ በሚጀምረበት ወቅት የማህፀን በር ጫፍ ካንሰር በመባል የታወቃል፡፡
- አብዛኛውን የማህፀን በር ጫፍ ካንሰር የሚያስከትለው ሂዩማን ፓፒሎማ ቫይረስ የሚባለው ኢንፌክሽን ነው፡፡
- ይህ ቫይረስ አብዛኛውን ጊዜ ሴቶችን ከያዛቸው በኀላ ወደካንሰርነት ሳይቀየር በእራሱ ጊዜ ይጠፋል፡፡ ኖም ይህ ኢንፌክሽን አንዳንድ ጊዜ በማህፀን በር ጫፍ ላይ የሚገኙ ሕዋሳት ላይ ጤናማ ያልኑ ለውጦችን ሊያስከትል ይችላል፡፡
- እነዚህ የቅድመ ካንሰር ለውጦች በጊዜ ያልታከሙ እንደነ ወደ ማህፀን በር ጫፍ ካንሰርነት ሊቀየሩ ይላሉ፡፡
- የማህፀን በር ጫፍ ካንሰር ከፍተኛ ህመም፤ በሎም ለሞት ሊዳርግ ይችላል፡፡

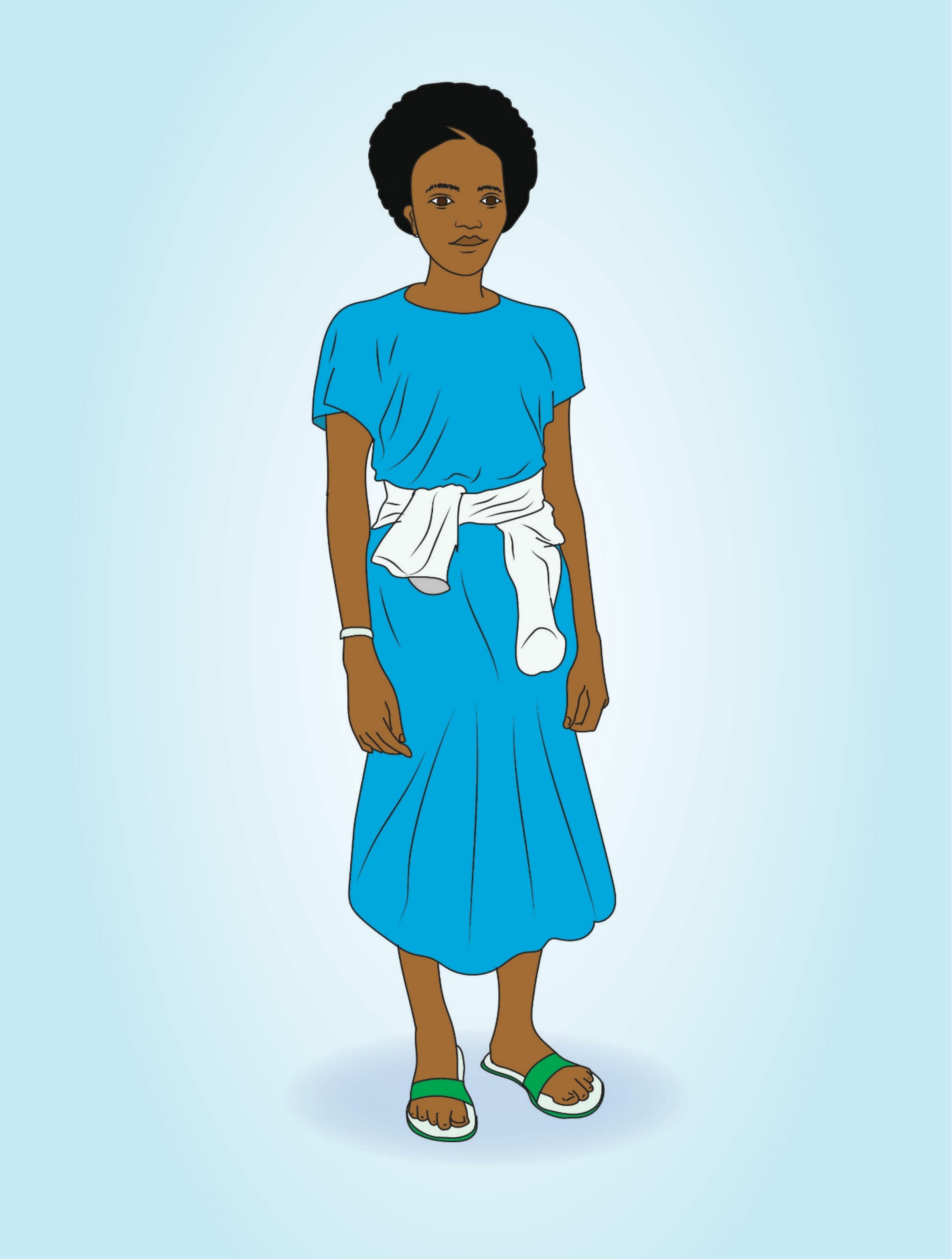

ይህች የምትመለከቷቱት ሴት ጤናማ ትመስላለች?

- ይህች የምትመለከተዋት ሴት ጤናማ ትመስላለች?

- የማህፀን በር ጫፍ ካንሰር ቀስ እያለ ስለሚሰራጭ አንዲት ሴት ስረጭቱ በሚጀምርበት ወቅት ምንም አይነት የተለየ ስሜት ላይኖራት ይችላል፡፡

- ቅድመ ካንሰር በሚጀምርበት ወቅት ሴቶች ምንም አይነት ሰሜት ላይሰማቸው ይችላል፤ምንም አይነት የህመም ወይንም የመድማት ምልክት ላይኖር ይችላል፡፡

- **ምንም እንክዋን የጤናማነት ስሜት ቢኖረንም እነዚህ የቅድመ ካንሰር ለውጦች ግን ሊኖሩ ይችላሉ፡፡**

በማህፀን በር ጫፍ ካንሰር ተጠቂ ከመን የምንከላከልባቸው ውጤታማ መንገዶች

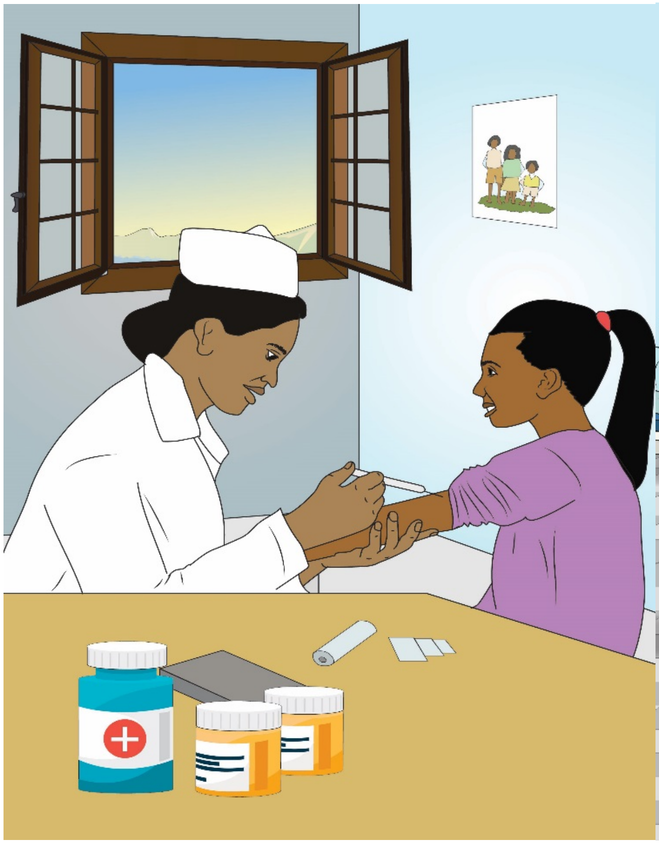

የኤች.ፒ. ቪ ቫይረስ ክትባት

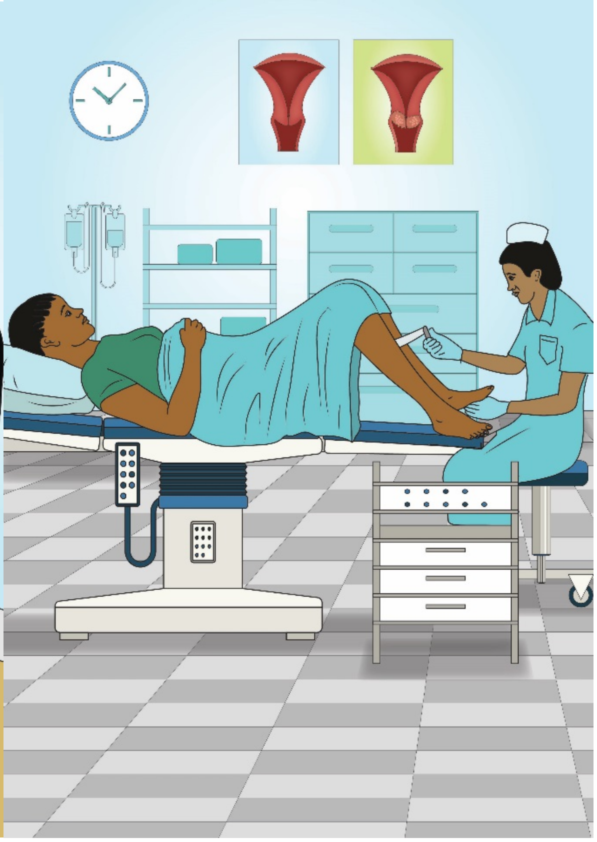

የማህፀን በር ጫፍ ካንሰር ምርመራ

አሁኑኑ እራስዎንም ነ የቤተሰብ አባላትን በማህፀን በር ጫፍ ካንሰር ተጠቂ ከመን የሚከላከሉበቸው ውጤታማ መንገዶች አሉ፡፡

- የማህፀን በር ጫፍ ካንሰርን ልንከላከል የምንችልበት ዋናው መንገድ በኤች.ፒ. ቪ ሊከሰት የሚችለውን ኢንፌክሽን በመከላከል ነው፡፡ ይህም ሴት ልጆች ምንም አይነት የግበረ ስጋ ግንኙነት ማድረግ ከመጀመራቸው በፊት የመከላከያ ክትባት በመስጠት ነው፡፡ እድሜያቸው ከ9-13 የኑ ሴት ልጆች በሙሉ ይህንን ክትባት ሊከተቡ ይገባል፡፡
- **ክትባቱን የወሰዱ ሴት ልጆች በማህፀን በር ጫፍ ካንሰር የመጠቃት እድላቸው በጣም አነስተኛ ነው፡፡**
- ሌላው ውጤታማ የመከላከያ መንገድ ደግሞ የማህፀን በር ጫፍ ላይ ያሉ የህዋስ ለውጦች ወደ ካንሰርነት የመቀየር እድላቸውን የሚያሳይ ልዩ ምርመራ በማድረግ ነው፡፡
- በማህፀን በር ጫፍ ላይ ለውጦች የታዩ እንደነ፤ እነዚህ ለውጦች ወደ ካንሰርነት እንዳይቀየሩ ለመከላከል የሚያስችል ህክምና ማግኘት ይችላሉ
- በአሁኑ ወቅት የጎንደር ዩንቨርስቲ እራስዎ የኤች.ፒ.ቪ ኢንፌክሽን ምርመራ ለማድረግ የሚያስችልዎ ጥናት እያካሄደ የሚገኝ ሲን ይህም ምርመራ በማህፀን በር ጫፍ ላይ የሚገኙ የህዋስ ለውጦች ካሉ በፍጥነት ተጨማሪ ምርመራ እንዲያደርጉ የሚያመላክት ይናል ፡፡

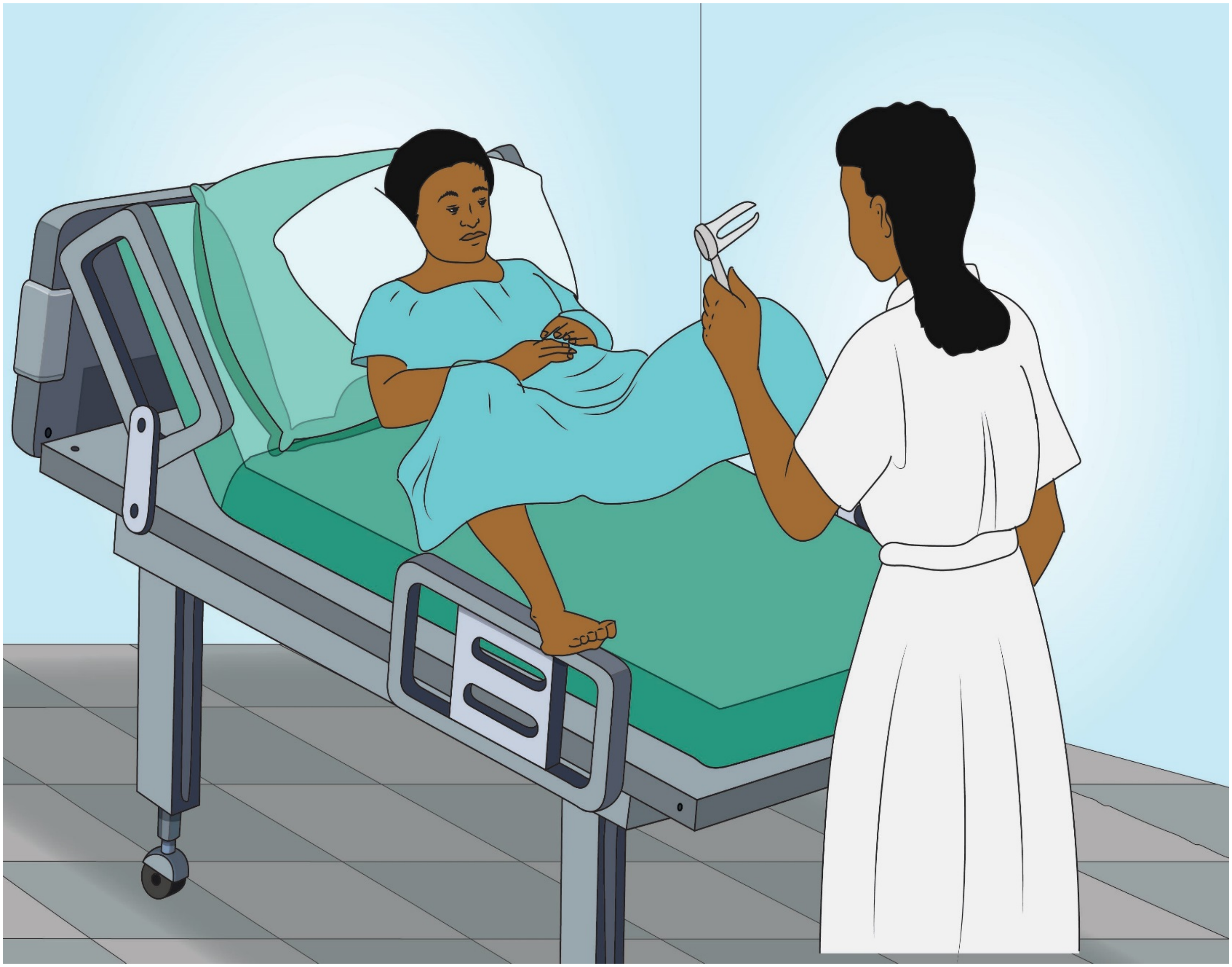

በዚህ ምስል ላይ ምን እየተካሄደ ያለ ይመስላችኋል?

- አንድ ዶክተር፤ ነርስ ይንም አዋላጅ ነረስ ወደብልት ዉስጥ በመመልከት የማህፀን በር ጫፍ ጤናማነትን ለማየት እና ለማረጋገጥ ይችላሉ፡፡
- ምረመራው ትንሽ ምቾትን የማሳጣት ስሜት ቢኖረውም ምንም አይነት ጉዳት የማያስከትል ሲን በጣም ውጤታማ ነው፡፡
- በጤና ማዕከል ውስጥ የሚገኝ አንድ ዶክተር፤ ነርስ ወይንም አዋላጅ ነረስ የኮምጣጤ ምርመራ የሚባል ፈጣን እና ቀለል ያለ የመርመራ አይነት ያደርጋሉ፡፡ ፈጣን የተባለበት ምክንያት ውጤቱን ወዲያዉኑ ማግኘት የሚቻል በመኑ ነው፡፡
- የጤና ባለሙያዋ/ው በዝግታ ወደብልት ውስጥ ሰርጥማያ ያስገባል/ ታስገባለች፡፡ *(ወደምስሉ ያመላክቱ)*
- የማህፀን በር ጫፍን በኮምጣጤ በማጠብ፤ ሳይታከሙ ቢቀሩ ወደ ካንሰርነት ሊለወጡ የሚቾሉ ነጭ ነጣጥቦች መኖራቸዉን ታጣራለች፡፡

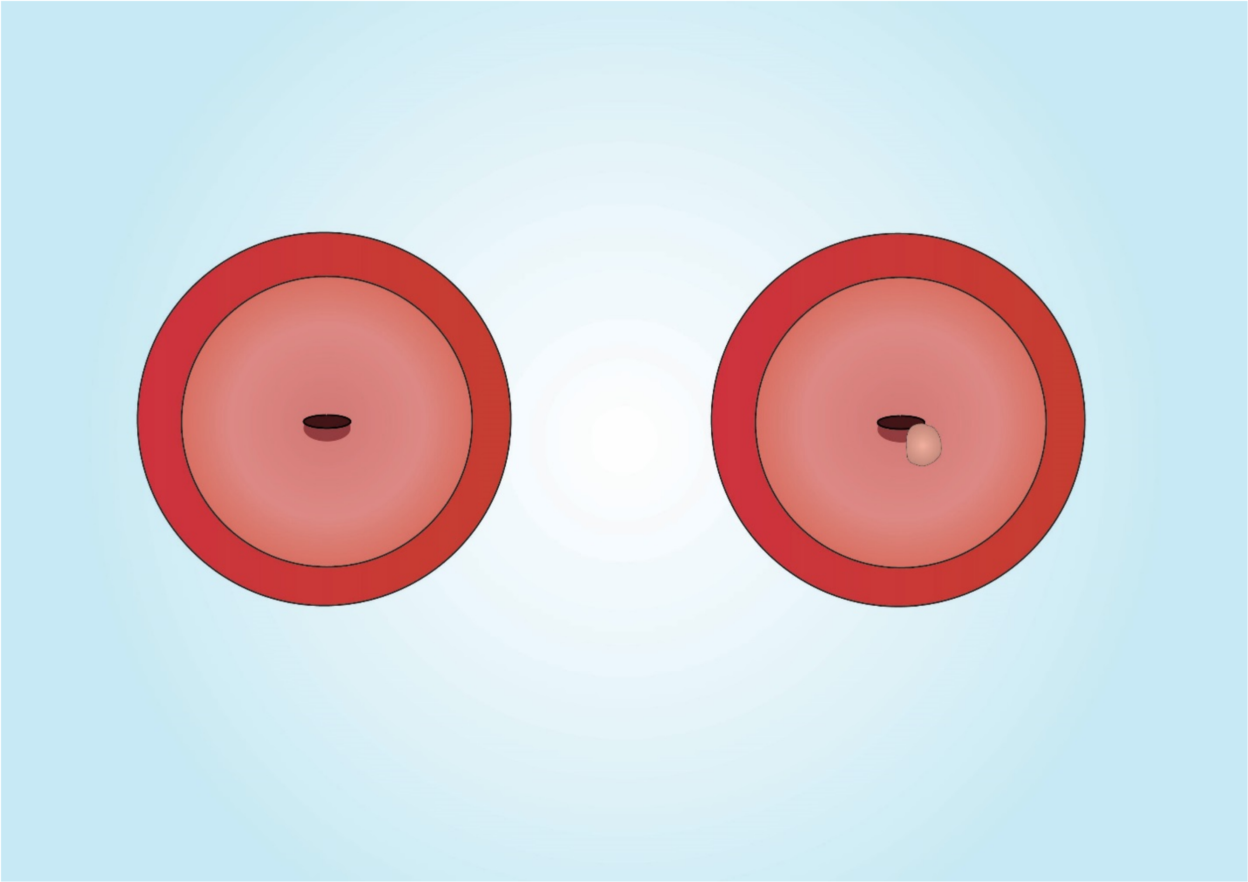

በዚህ ምስል ላይ ምን ይመለከታሉ?

- ይህ ምስል አንድ ዶክተር፤ ነርስ ይንም አዋላጅ ነረስ የማህፀን በር ጫፍ ጤናማነትን በሚመረምሩበት ወቅት የሚኖራቸውን ዕይታ ያመላክታል፡፡
- በመጀመሪያው ምስል ላይ የምንመለከተው ጤናማ የነ የማህፀን በር ጫፍ ነው፡፡
- በሁለተኛው ምስል ላይ የምንመለከተው እላዩ ላይ ነጭ ነጥብ ያረፈበት ወይንም "ቅድመ ካንሰር" መኖሩን የሚያመላክት የማህፀን በር ጫፍ ነው፡፡

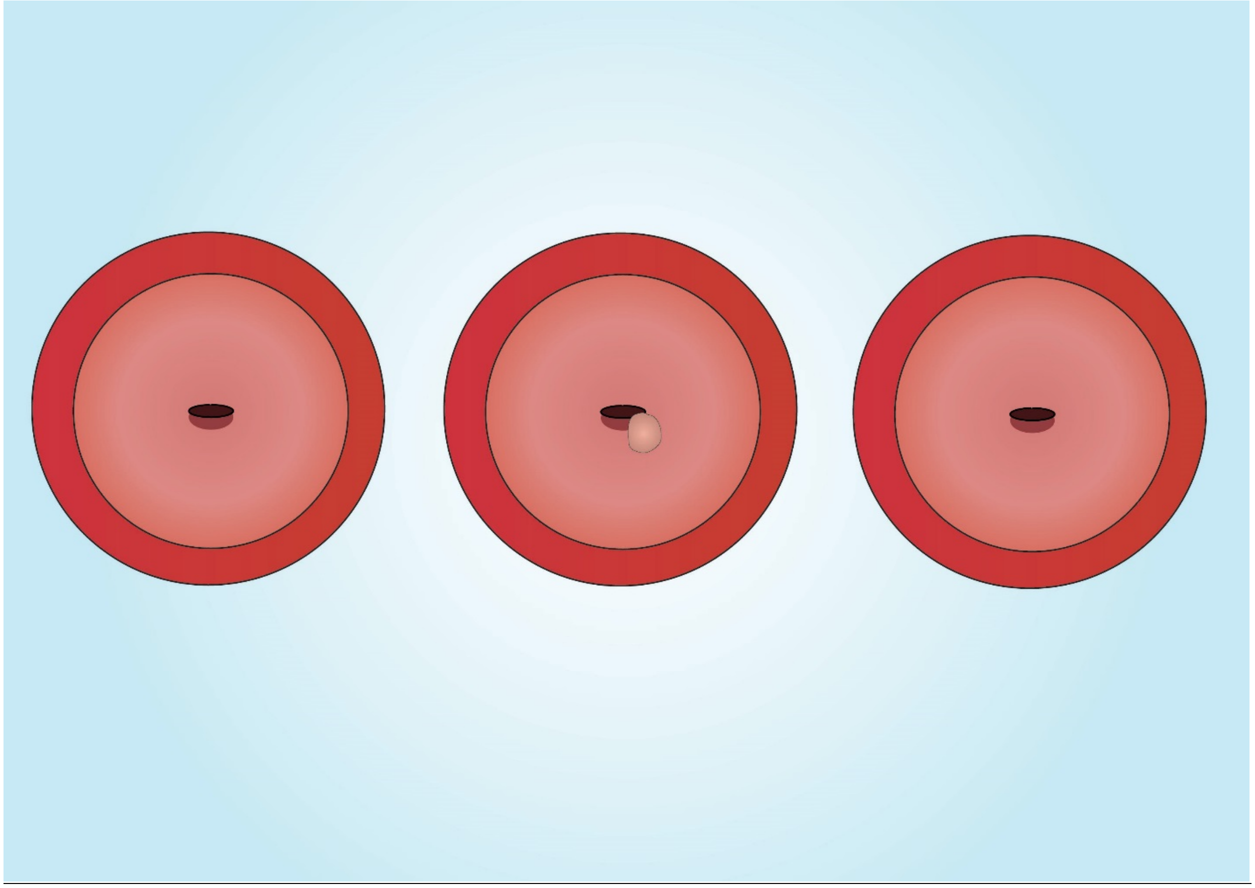

(Terms to be added)

ይህንን ምስል ለየት የሚያደርገው ምንድን ነው?

- እነዚህ ነጭ ነጥቦች ወደካንሰርነት ከመለወጣቸው በፊት ለማስወገድ የሚያችል እና ቀላል የነ ህክምና አለ፡፡
- አንድ ዶክተር ወይንም ነርስ ይህንን ጤናማ ያልነ ነጥብ በበረዶ በማቀዝቀዝ ለማስወግድ ይችላል፡፡
- ምርመራውን ከሚያደርጉ ሴቶች መካከል በአብዛኛው ይህ ተጨማሪ ሕክምና አያስፈልጋቸውም፡፡
- ይህ ህክምና አስፈላጊ ኖ የተገኘ እንደነ ግን ምንም አይነት ጉዳት የማያደርስ ሲን የማህፀን በር ጫፍ ካንሰር እነዳያድግም ይከላከላል፡፡
- **ቅድመ ካንሰር ቀደም ብሎ በሰውነት ውስጥ መኖሩ ከታወቀ በጣም በቀላሉ ማከም እና ማዳን ይቻላል፡፡**
- አልፎ አንዳነድ ሴቶች ተጨማሪ ህክምና እንዲያገኙ ወደ ሌላ የጤና ጣቢያ ወይንም ስፒታል የሚላኩበት አግባብ ሊኖር ይችላል፡፡
- አገልግሎት የሚሰጥዎት ዶክተር፤ ነርስ ይንም አዋላጅ ነረስ የሚያስፈልግዎትን ህክምና እና እንክብካቤ እንዲያገኙ ያደርጋሉ፡፡

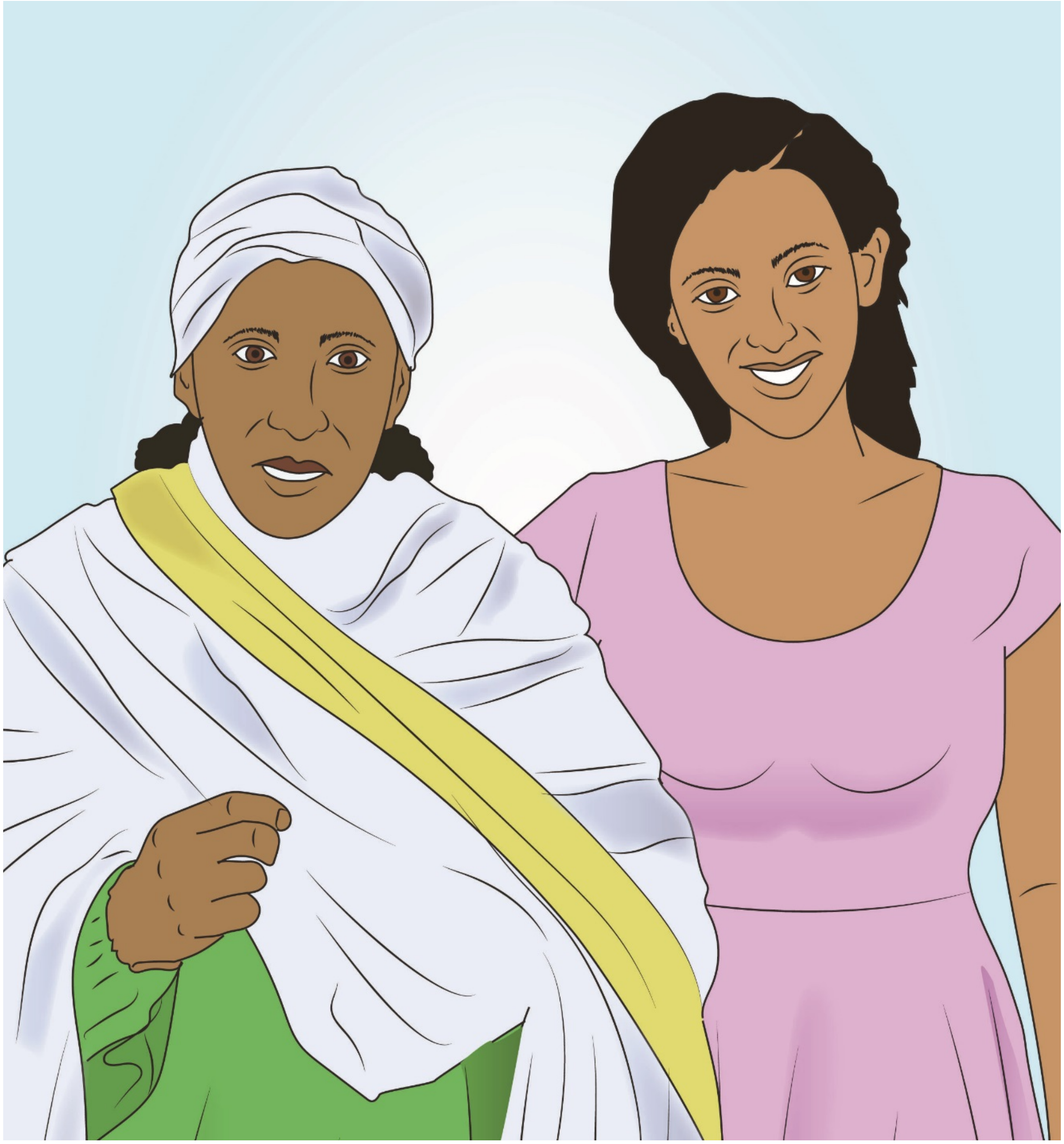

- ጎንደር ዩንቨርሲቲ የኤች.ፒ.ቪ ኢንፌክሽን በውስጥዎ መኖር/ አለመኖሩን የሚያረጋግጥ ልዩ ምረመራ የጥናቱ አንድ አካል በማድረግ አመቻችቷል፡፡
- ይህ ምርመረም እራስዎት የተዘጋጀልዎትን ልዩ ብሩሽ በመጠቀም በማህፀን በር ጫፍ ላይ የሚገኙ ሕዋሳትን እንዲወስዱ የሚያስችል ነው፡፡
- ከማህፀን በር ጫፍ ላይ የተወሰዱት ሕዋሳት በተዘጋጀው ብልቃጥ ውስጥ ተከተው ለምርመራ ወደ ቤተሙከራ ይላካሉ፡ ፡ ውጤቱ ከ6-8 ሳምንት ባለ ጊዜ ውስጥ ይደርሳል፡፡
- ***ትክክለኛውን ፅሁፍ ከተዛማጅ የጥናቱ ክንድ ጋር በማጣመር የሚነበብ፡-*** ይህንን ምርመራ (በቤትዎ ***(ክንድ 1)***)፣ (በጤና ኬላ ***(ክንድ 2)***)፣ (በጤና ጣቢያ ***(ክንድ 3)***) ማድረግ ይችላሉ፡፡
- የኤች.ፒ.ቪ ምርመራ ውጤትዋዎ ፖዘቲቭ (አዎንታዊ) ከነ ፣ በማህፀን በር ጫፍ ካንሰር የመጠቃት ዕድልዎ ከፍተኛ ስለሚን ወዲያውኑ የኮምጣጤ ምርመራ የሚባለውን ማድረግ እንደሚያስፈልግዎት ያመላከታል፡፡
- የኤች.ፒ.ቪ ምርመራ ውጤትዋዎ ኔጋቲቭ (አሉታዊ) ከነ ፣ በማህፀን በር ጫፍ ካንሰር የመጠቃት ዕድልዎ ዝቅተኛ ስለሚን ወዲያውኑ የኮምጣጤ ምርመራ የሚባለውን ማድረግ እንደማያስፈልግዎት ያመላከታል፡፡ የኤች.ፒ.ቪ ምርመራ ልክ በ5 አመቱ በድጋሚ እነዲያደረጉ ይመከራል፡፡

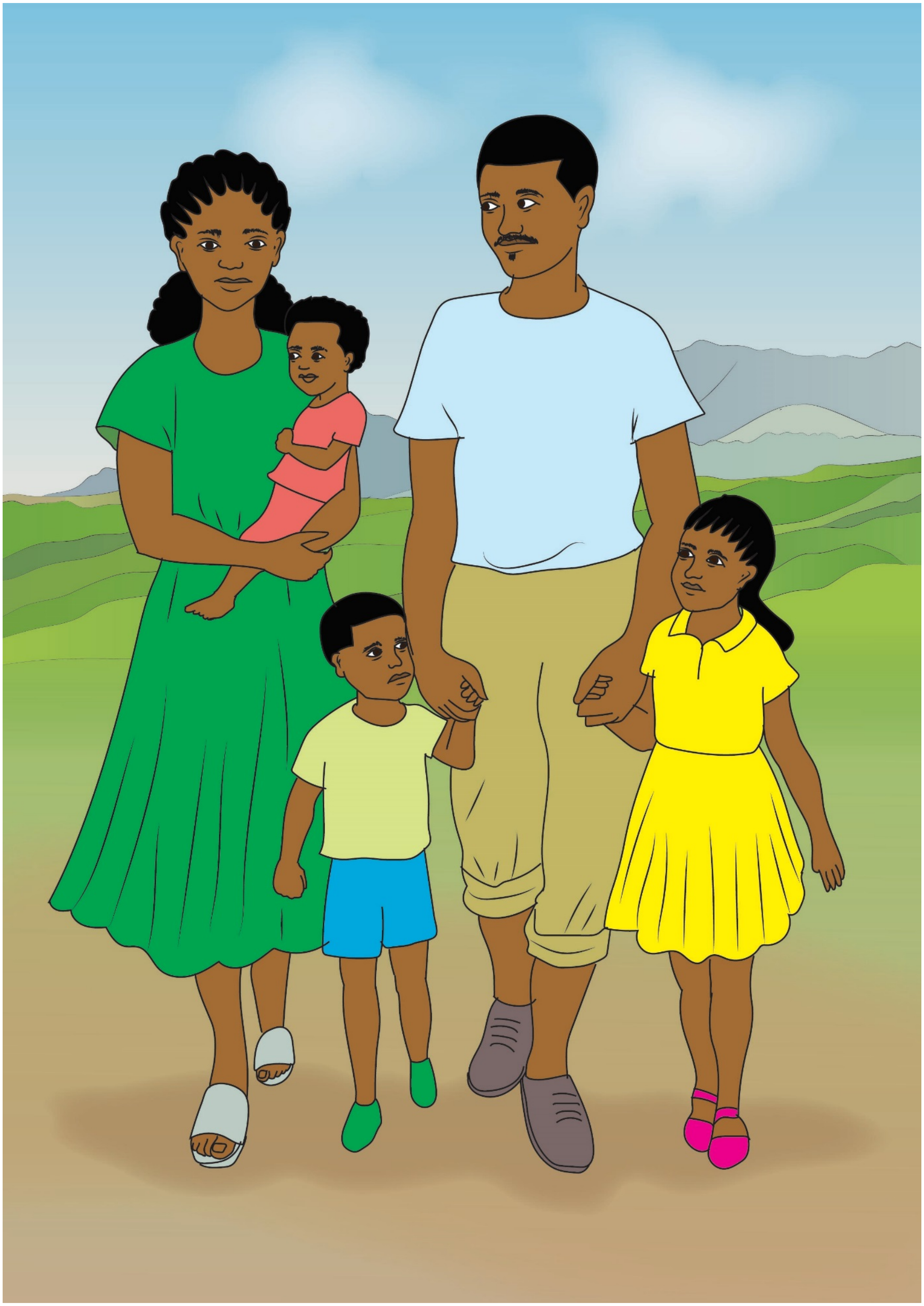

በዚህ ምስል ላይ ምን ይመለከታሉ?

ይህ የምትመለከቱት ቤተሰብ ደስተኛ እና ጤናማ ቤተሰብ ይመስላል?

በዚህ ምስል ላይ ያለችው ሴት ብትታመም ቤተሰቡን ምን ሊያጋጥመው ይችላል?

- ይህ የምትመለከቱት ምስል የአንድ ጤናማ እና ደስተኛ ቤተሰብ ምስል ነው፡፡
- አንዷት ሴት በማህፀን በር ጫፍ ካንሰር በመያዝ ስትታመም ወይንም ህይወተዋ ሲያልፍ ብዙ ሰዎች በእሰዋ ህመም ወይንም ሞት ሳቢያ ይጎዳሉ፡፡ ልጆቸዋ፤ ቤተሰብዋ እንዲሁም መላው ማህበረሰብ በማህፀን በር ጫፍ ካንሰር ምክንያት ጉዳት ይደርስበታል፡፡
- የሴት ጤናማነት ለመላው ቤተሰብ እነዲሁም ማህበረሰብ ጠቃሚ ስለነ፤ የማህፀን በር ጫፍ ካንሰርን መከላከል በጣም አስፈላጊ ነው፡፡

ቁልፍ መልዕክቶች

- የማህፀን በር ጫፍ ካንሰር ልንከላከል የምንችለው በሽታ ነው፡፡
- የኤች.ፒ.ቪ ኢንፌክሽን ወይንም የማህፀን በር ጫፍ ህዋስ ለውጦች መኖር ወይንም አለመኖራቸውን የሚያመላክት ቀላል ምርመራ አለ፡፡
- እነዚህ ምርመራዎች ምንም አይነት ጉዳት የማያስከትሉ እና ፈጣን ሲኑ ፤ አገልግሎቱን በ _________የጤና ጣቢያ ከክፍያ ነፃ ማግኘት ይቻላል፡፡
- ማንኛዋም ሴት ለማህፀን በር ጫፍ ካንሰር የተጋለጠች ናት፡፡ እድሜያቸው በ 30 እና በ49 መካከል የሚገኙ ሴቶች በሙሉ ይህንን ምርመራ ማድረግ ይኖርባቸዋል፡፡
- በማህፀን በር ጫፍ ህዋሳት ላይ ለውጦች መኖራቸው በጊዜ ከታወቀ ቀላል እና ምንም ጉዳት የማያስከትል ህክምና ማግኘት ይቻላል፡፡
- ለልጃገረዶች የሚሰጥ የማህፀን በር ጫፍ ካንሰርን ለመከላከል የሚያስችል ክትባት አለ፡፡

ዋና ነጥቦቹን በመከለስ ተሳታፊዎች ጥያቄዎች እንዳላቸው ይጠይቁ፡፡

ቁልፍ መልዕክቶች

- የማህፀን በር ጫፍ ካንሰር ልንከላከል የምንችለው በሽታ ነው፡፡
- የኤች.ፒ.ቪ ኢንፌክሽን ወይንም የማህፀን በር ጫፍ ህዋስ ለውጦች መኖር ወይንም አለመኖራቸውን የሚያመላክት ቀላል ምርመራ አለ፡፡
- እነዚህ ምርመራዎች ምንም አይነት ጉዳት የማያስከትሉ እና ፈጣን ሲኑ ፤ አገልግሎቱን በ _________ የጤና ጣቢያ ከክፍያ ነፃ ማግኘት ይቻላል፡፡
- ማንኛዋም ሴት ለማህፀን በር ጫፍ ካንሰር የተጋለጠች ናት፡፡ እድሜያቸው በ 30 እና በ49 መካከል የሚገኙ ሴቶች በሙሉ ይህንን ምርመራ ማድረግ ይኖርባቸዋል፡፡
- በማህፀን በር ጫፍ ህዋሳት ላይ ለውጦች መኖራቸው በጊዜ ከታወቀ ቀላል እና ምንም ጉዳት የማያስከትል ህክምና ማግኘት ይቻላል፡፡
- ለልጃገረዶች የሚሰጥ የማህፀን በር ጫፍ ካንሰርን ለመከላከል የሚያስችል ክትባት አለ፡፡

እራሳችንን፤ እናቶቻችንን እና ሴት ልጆቻችንን በማህፀን በር ጫፍ ካንሰር ከመጠቀት እንከላከል፡፡ እንመረመር/ የጤናችንን ሁኔታ እንወቅ/ ጤንነታችንን እንፈትሽ!

(To be graphically adjusted and the term “get screened” to be selected among the three terms)

እራሳችንን፤ እናቶቻችንን እና ሴት ልጆቻችንን በማህፀን በር ጫፍ ካንሰር ከመጠቃት እንከላከል፡፡ እንመረመር/ የጤናችንን ሁኔታ እንወቅ/ ጤንነታችንን እንፈትሽ!

(To be graphically adjusted and the term “get screened” to be selected among the three terms)

(Logos to be changed upon recommendation and designed as per requirements of the schools)

የጥናቱ መግቢያ

- ንግግራችንን እስከአሁን በጥሞና ስላዳመጣችሁን እናመሰግናለን፡፡
- አሁን ደግሞ “እናቶቻችንን እና ሴት ልጆቻችንን በማህፀን በር ጫፍ ካንሰር ከመጠቃት እንከላከል” በሚል ርዕስ በጎንደር ዩንቨርስቲ እና ቶሮ ዩንቨርስቲ ካሊፎርንያ ከየአካባቢው የቀበሌ አመራሮች በተገኘው ፈቃድ መሰረት እያካሄዱ ስለሚገኙት ምርምር እና ጥናት እነግራችኋለሁ፡፡
- ይህ ምርምር ሴቶችን እንዴት ስለማህፀን በር ጫፍ ካንሰር ማስተማር እነደሚገባ እና የተሻለ የማህፀን በር ጫፍ ካንሰር የምርመራ መንገድ የትኛው እንደነ   እያጠና ይገኛል፡፡
- ይህንን ጥናት የሚያካሄዱ ተመራማሪዎች ሴቶች የኤች.ፒ.ቪ ምርመራ (በመኖሪያ ቤታቸው፤ በጤና ጣቢያ፤ጤና ኬላ) ካደረጉ በኋላ የተዘጋጀውን መጠይቅ በመመለስ በምርመራው ወቅት የነበራቸውን ተሞክሮ ለማወቅ ጥረት ያደርጋሉ፡፡

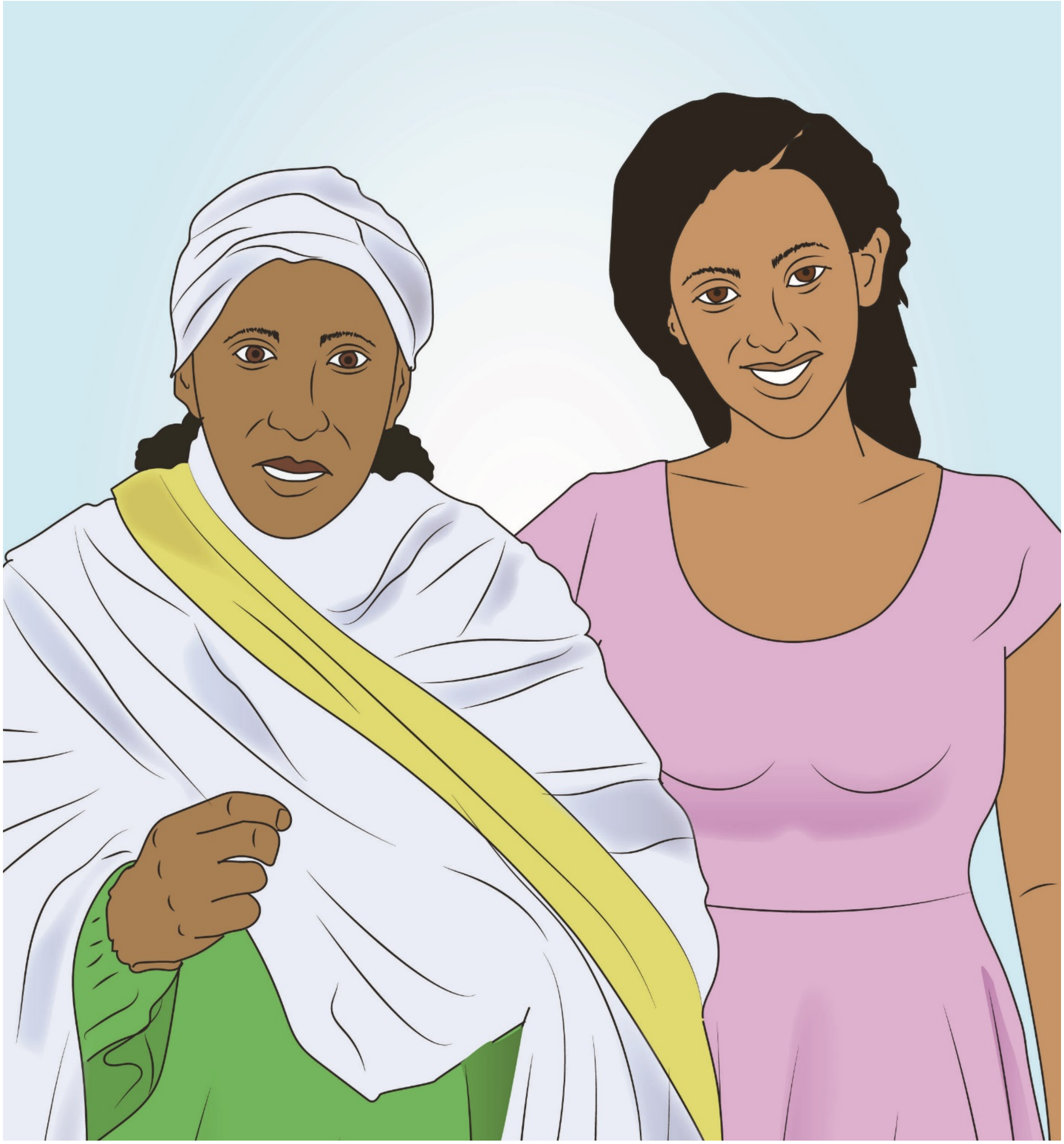

- እናንተም በእዚህ ጥናቱ በሚከናወንበት ማህበረሰብ ውስጥ ነዋሪ እንደመናችሁ፤ በጥናቱ እንድትሳተፉ በአክብሮት እንጠይቃለን፡፡

- በዚህ ጥናት መሳተፍዎ በሙሉ ፈቃደኝነትዎት ላይ መሰረት ያደረገ ሲን ፤ በሚያገኙዋቸው የህክምና አገልግሎቶች ላይ ምንም አይነት ተፅእኖ አያሳድርም፡፡

- በዚህ ጥናት ለመሳተፍ ፈቃደኛ የኑ እንደነ ውጤትዎንም ነ የሚሰጡንን ማንኛዉም መረጃ በሚስጢር የምንይዝ ይናል፡፡ ስምዎትም አይመዘገብም፡፡

- ከምርመራው በኋላ እኛን ለማነጋገር ጊዜዎትን መስዋት ማድረግዎትን ለማመስገን ያህል የፀጉር ቅባት/ ሳሙና በስጦታነት የምናበረክትልዎ ይናል፡፡ ምርመራው ቃለ-መጠይቁን ጨምሮ ወደ አንድ ሰአት ገደማ የሚወስድ ይናል

- በዚህ ጥናት ውስጥ ለመሳተፍ እድሜዎ ከ 30-50 አመት ባለው ውስጥ መን ሲኖርበት፤ ከዚህ በፊት የመህፀን በር ጫፍ ካንሰር ምርመራ አድርገው የማያውቁ ሊን ይገበል፡፡ በአሁኑ ወቅት አርጉዝ የኑ ወይንም ማህፀንዎን ያስወጡ እንደነ በዚህ ጥናት ለመሳተፍ አይችሉም፡፡

- አሁን እየጠየቅሁ ያለሁት በዚህ ጥናት ላይ ለመሳተፍ ፈቃደኛ መንዎን ነው፡፡ ለመሳተፍ ፈቃደኛ ከኑ ይህንን ስብሰባ ከጨረስን በኋላ በገል እንድንነጋገር ወደኋላ በመቅረት እንድትጠብቁኝ በትህትና እጠይቃለሁ፡፡

እናቶቻችን እና ሴት ልጆቻችን በማህፀን በር ጫፍ ካንሰር እንዳይጠቁ መከላከል

ለማህፀን በር ጫፍ ካንሰር የግል/ የራስን ናሙና አወሳሰድ መመሪያ

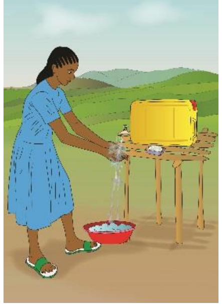	ናሙና ከመውሰድዎ በፊት፡ እጅዎን በሰሙና እና በውሃ በደንብ ይታጠቡ
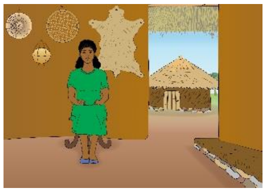	ናሙናውን በመኖሪያ ቤትዎ የሚወስዱ ከሆነ ገለልተኛ እና ልብስ ለማውለቅ የሚመቸዎትን ቦታ ይምረጡ፡፡ የውስጥ ልብስዎን ያውልቁ::
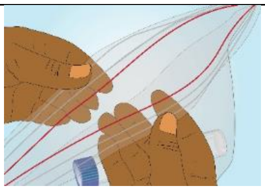	የናሙና መውሰጃ ብልቃጥ፤ ብሩሽ እና መመሪያ የያዘውን ላስቲክ ይክፈቱት
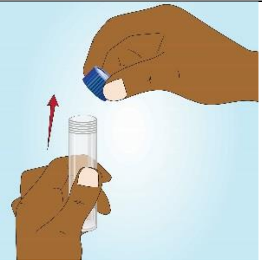	ብልቃጡን ከላስቲኩ በማውጣት ክዳኑን ይክፈቱ፡፡
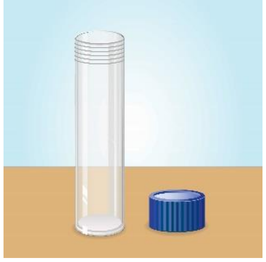	የብልቃጡን ክዳን ከከፈቱ በኋላ ወድቆ በውስጡ የሚገኘው ፈሳሽ እንዳይደፋ በማያወላውል ወለል ላይ ያስቀምጡት፡፡

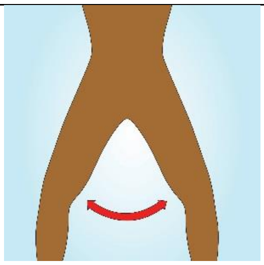	ናሙናውን ለመውሰድ ምቹ የሆነ ቁመና ይፈልጉ/ይያዙ፡፡ አብዛኛው ሴቶች ቆሞ እግርን በመክፈት ናሙና መውሰድ የተሻለ ምቾት እንደሚሰጥ ይገልፃሉ፡፡
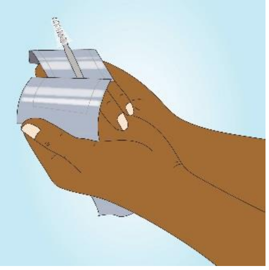	ናሙና የመውሰጃ ብሩሽ የያዘውን መጠቅለያ ፕላስቲክ በጫፉ ላይ የሚገኘውን ክፍተት በመያዝ ይክፈቱ፡፡<br>ከከፈቱት በኋላ የብሩሹን ፀጉሮች በእጅዎ ወይንም ሌላ አካል ሳይነካቸው እና ሳይበከሉ ከላስቲኩ ብሩሹን ያውጡት፡፡
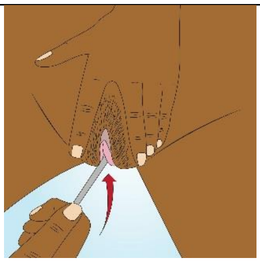	ብሩሹን በዝግታ ወደ ብልት ውስጥ መግባት እስከሚያቆም ድረስ ያስገቡት፡፡<br>ለመግባት ያስቸገርዎት ከሆነ፤ ለመግባት እስኪችል ድረስ ቀስ በቀስ ያሽከርክሩት፡፡
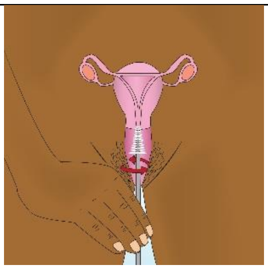	አንዴ ብሩሹ ከገባ በኋላ ከ3-5 ጊዜ በዝግታ ያሽከርክሩት፡፡ ብሩሹን በጥንቃቄ ከብልት ውስጥ ያውጡት፡፡
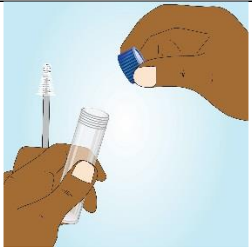	ብሩሹን በአንድ እጅዎ እንደያዙ ብልቃጡን ያንሱት፡፡ በውስጡ ያለው ፈሳሽ እንዳይደፋ ይጠንቀቁ፡፡
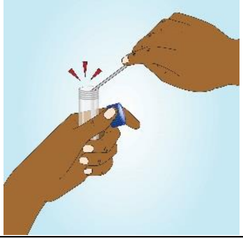	ናሙና የወሰዱበትን ብሩሽ ወደ ብልቃጡ በመመለስ በብሩሹ ዱላ ላይ አፈንግጦ በሚታየው ቦታ ላይ የብልቃጡን ጠርዝ በማስደገፍ የብሩሹን ዱላ ይስበሩት፡፡

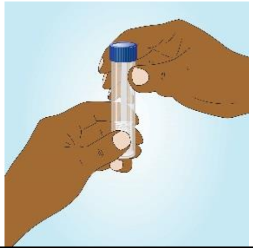	የብልቃጡን ክዳን መልሰው ይዝጉት፡፡ ፍሳሽ እንዳይኖር በደንብ መዘጋቱን ያረጋግጡ፡፡
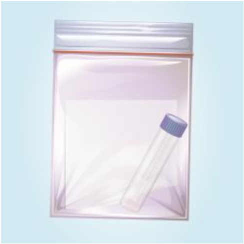	ብልቃጡን እንዲሁም ቆሻሻውን (የብሩሹን መጠቅለያ እና የብሩሹን ዱላ ስበሪ) መልሰው ወደ ላስቲኩ ውስጥ ያስገቡ፡፡ ላሰቲኩን መጀመሪያ ለሰጠዎት የጤና ባለሙያ በ24 ሰዐት ውስጥ መልሰው ያስረክቡ፡፡
